# A central region in the minor capsid protein of papillomaviruses facilitates viral genome tethering and membrane penetration for mitotic nuclear entry

**DOI:** 10.1371/journal.ppat.1006308

**Published:** 2017-05-02

**Authors:** Inci Aydin, Ruth Villalonga-Planells, Lilo Greune, Matthew P. Bronnimann, Christine M. Calton, Miriam Becker, Kun-Yi Lai, Samuel K. Campos, M. Alexander Schmidt, Mario Schelhaas

**Affiliations:** 1 Cellular Virology, Institutes of Molecular Virology and Medical Biochemistry, Center for Molecular Biology of Inflammation (ZMBE), University of Münster, Münster, Germany; 2 Cells in Motion, CiM, Cluster of Excellence EXC, Münster, Germany; 3 Institute of Infectiology, Center for Molecular Biology of Inflammation (ZMBE), University of Münster, Münster, Germany; 4 Department of Immunobiology, University of Arizona, Tucson, AZ, United States of America; 5 BIO5 Institute, University of Arizona, Tucson, AZ, USA; 6 Department of Molecular and Cellular Biology, University of Arizona, Tucson, AZ, United States of America; 7 Cancer Biology Graduate Interdisciplinary Program, University of Arizona, Tucson, AZ, United States of America; Penn State University School of Medicine, UNITED STATES

## Abstract

Incoming papillomaviruses (PVs) depend on mitotic nuclear envelope breakdown to gain initial access to the nucleus for viral transcription and replication. In our previous work, we hypothesized that the minor capsid protein L2 of PVs tethers the incoming vDNA to mitotic chromosomes to direct them into the nascent nuclei. To re-evaluate how dynamic L2 recruitment to cellular chromosomes occurs specifically during prometaphase, we developed a quantitative, microscopy-based assay for measuring the degree of chromosome recruitment of L2-EGFP. Analyzing various HPV16 L2 truncation-mutants revealed a central chromosome-binding region (CBR) of 147 amino acids that confers binding to mitotic chromosomes. Specific mutations of conserved motifs (IVAL286AAAA, RR302/5AA, and RTR313EEE) within the CBR interfered with chromosomal binding. Moreover, assembly-competent HPV16 containing the chromosome-binding deficient L2(RTR313EEE) or L2(IVAL286AAAA) were inhibited for infection despite their ability to be transported to intracellular compartments. Since vDNA and L2 were not associated with mitotic chromosomes either, the infectivity was likely impaired by a defect in tethering of the vDNA to mitotic chromosomes. However, L2 mutations that abrogated chromatin association also compromised translocation of L2 across membranes of intracellular organelles. Thus, chromatin recruitment of L2 may in itself be a requirement for successful penetration of the limiting membrane thereby linking both processes mechanistically. Furthermore, we demonstrate that the association of L2 with mitotic chromosomes is conserved among the alpha, beta, gamma, and iota genera of *Papillomaviridae*. However, different binding patterns point to a certain variance amongst the different genera. Overall, our data suggest a common strategy among various PVs, in which a central region of L2 mediates tethering of vDNA to mitotic chromosomes during cell division thereby coordinating membrane translocation and delivery to daughter nuclei.

## Author summary

Papillomaviruses can cause carcinogenic malignancies such as cervical cancer. Like most DNA viruses, papillomaviruses must deliver their genome to the cell nucleus during initial infection, where it is expressed and replicated. However, papillomaviruses make use of unconventional mechanisms for genome delivery. They reside on the cell surface for protracted, hour-long times, before they are taken up by a novel endocytic mechanism. Moreover, they are delivered to the *trans*-Golgi-network by non-canonical endosomal trafficking prior to nuclear delivery. For entry into the nucleus, papillomaviruses access the nuclear space after nuclear envelope breakdown during mitosis unlike most other intranuclear viruses. The detailed mechanism how the viral genome is directed to nascent nuclei during mitosis remains elusive. Our previous work suggested that the minor capsid protein L2 may tether the incoming viral genome to mitotic chromosomes to direct it to the nascent nuclei. This work identifies a conserved central region in L2 protein to be necessary and sufficient for tethering. Moreover, it demonstrates that this mechanism is conserved across different papillomavirus genera. Importantly, this report also provides evidence that the processes of nuclear import by tethering and membrane penetration are mechanistically linked.

## Introduction

Over 170 human papillomavirus (HPV) types and numerous PV types from vertebrate animals constitute the family of *Papillomaviridae* that contains 39 distinct genera [1]. PVs infect skin, oral and anogenital epithelia, where they either persist asymptomatically or cause neoplasia with a range of malignancies. For example, the HPV types 16 and 18 account for more than half of the cervical carcinoma cases worldwide [2, 3].

Like most DNA viruses, PVs need to deliver their genome to the host cell nucleus to initiate viral transcription and replication. For this, most intranuclearly replicating viruses use the nuclear import machinery of the host cell (reviewed in [4]). As an exception, HPVs depend on mitosis for nuclear entry during which the viral DNA (vDNA) gains access to the nuclear space upon nuclear envelope breakdown (NEBD) [5, 6]. In line with the restriction of PV entry into dividing cells, PVs enter the basal stem cells or transit-amplifying cells in squamous epithelia during initial infection [7, 8].

PVs are non-enveloped DNA viruses with an icosahedral (T = 7d) capsid of about 55 nm in diameter. The capsid is formed by 72 pentamers of the major capsid protein L1, and contains up to 72 copies of the minor capsid protein L2 [9, 10]. The encapsidated, circular, double-stranded DNA genome of about 8-kb is chromatinized with cellular histones [11]. Since the HPV life cycle and thus progeny virus production require epithelial differentiation [12], so-called pseudoviruses (PsVs) are typically used as a surrogate to study initial infection [13]. PsVs are composed of L1/L2 capsids and a pseudogenome, e.g. a plasmid encoding a reporter gene that indicates successful entry upon expression.

Upon initial binding of the HPV16 particle to heparan sulphate proteoglycans at the cell surface or in the extracellular matrix, a series of extracellular conformational changes are triggered in the viral capsid [14–20]. These structural alterations appear to prime the virus capsid for engagement of an elusive secondary receptor that triggers endocytosis (discussed in [21]). HPV16, 18 and 31 are endocytosed by a clathrin-, caveolin-, lipid raft-, and dynamin-independent pathway that depends on actin dynamics [22, 23]. The virus traffics through endosomal compartments, where the major capsid protein L1 dissociates from a subviral complex formed by L2 and vDNA (L2/vDNA) [22, 24–26]. This subviral complex is routed to the *trans*-Golgi network (TGN) via retrograde pathways, to eventually reach the nucleus, where it localizes to PML nuclear bodies (PML-NBs) [24, 27, 28]. Further, the subviral complex requires mitotic NEBD to gain access to the nuclear space [5]. However, how the subviral complex penetrates limiting membranes and is recruited to mitotic chromatin, and how it is retained in the nucleus following mitosis is not understood mechanistically.

The PV minor capsid protein L2 is a multifunctional protein that exerts crucial roles during virus entry as well as during virus assembly (reviewed in [29]). The L2 protein sequence can be divided into N-terminal, central, and C-terminal domains according to their functions. For assembly of progeny virions, newly synthesized L2 is imported into and retained within the nucleus via its N- and C-terminal nuclear localization signals (NLSs) and a central arginine-rich nuclear retention signal (NRS), respectively [30, 31]. In the nucleus, L2 likely packages vDNA into assembling capsids through its N- and C-terminal DNA-binding sites, and through interaction of its C-terminal domain with L1 in the nucleus [32–35].

During PV entry, L2 is important for a number of steps that guide the vDNA to the site of replication. Exposure of the L2 N-terminus to the capsid surface through interaction with cyclophilins and cleavage by extracellular furin are required for infectious cell entry to potentially facilitate the interaction with the elusive secondary receptor [14, 19, 21]. Furthermore, L2 is implicated in vesicular trafficking since domains in the central part of L2 interact with the cytosolic regulators of endosomal recycling SNX17 and SNX27 [36, 37]. L2 is also required for retrograde trafficking from early endosomes to the TGN through direct interaction of the L2 C-terminal part with the retromer [38].

L2 would need to be cytosolically exposed to interact with these cellular sorting factors. It remains unknown exactly when and how L2 spans across or penetrates the membranes of vesicular compartments to interact with the cytosolic cellular interaction partners. The N-terminal transmembrane (TM) domain and C-terminal membrane-destabilizing peptide have been implicated in translocation across membranes originally presumed to occur within endosomal compartments [39, 40]. Recently, it has been shown that L2 is capable of using the N-terminal TM domain to span across intracellular membranes during post-entry subcellular trafficking [41]. The orientation of L2 within membranes appears to be consistent with a type I topology. In this model, some or all of the L2 protein C-terminal of the TM domain would be cytosolic, whereas a small N-terminal portion would be luminal. This would place the SNX and retromer binding sites of L2 within the cytosol, explaining how L2 could recruit these cytosolic trafficking factors to facilitate endosomal and retrograde sorting of the vDNA.

It has been proposed that the vDNA remains within a vesicular compartment prior to and after passage through the TGN, where the vesicular vDNA would be tethered to mitotic chromosomes until the nascent daughter nuclei would have been formed [42]. In this model, it has been suggested that L2 commandeers microtubule and spindle transport systems to achieve localization to mitotic chromosomes. As the C-terminus of L2 is also capable of binding to the microtubular motor protein dynein, vesicular or free L2/vDNA may utilize microtubules to traverse the cytoplasm to facilitate TGN localization and achieve localization at the microtubule organizing center prior to nuclear entry [43, 44]. Upon entry into the nucleus, the C-terminal PML-NB localization domain and the central SUMO interacting motif of L2 may target L2/vDNA to PML-NBs, where L2 could facilitate the early transcription and replication events [28, 45, 46].

Given that L2 binds vDNA to escort it into the nucleus, and because L2 and vDNA associate with chromosomes during mitosis, we previously proposed that L2 tethers vDNA to mitotic chromosomes upon NEBD to facilitate the nuclear delivery of the PV genome during cell division [5, 27, 33]. In this study, we used quantitative fluorescence microscopy to identify the required and/or sufficient domains in L2 that mediate chromosomal association. When N-and/or C-terminally truncated HPV16 L2 proteins were tested for their association with mitotic chromosomes, a conserved, central region of 147 amino acids (residues 188–334) in HPV16 L2 conferred binding to chromosomes. Site-directed mutagenesis conserved residues within this newly defined region of 147 amino acids identified several chromosome-binding deficient L2 mutants. Importantly, PsVs containing chromosome-binding deficient L2 mutants were unable to deliver the vDNA to the nucleus. Interestingly, these mutants were also unable to exit vesicular compartments as determined by a novel L2-BirA translocation assay described in detail in [47]. Thus, our overall data indicated a nuclear entry strategy common to various tested PVs, in which a central conserved region in L2 mediated tethering of the vDNA to mitotic chromosomes thereby directing viral genomes to the nascent nuclei during cell division. Further, we propose that the ability to bind mitotic chromatin is functionally linked to translocation of the L2/vDNA complex across a post-Golgi limiting membrane.

## Results

### HPV16 vDNA and L2-EGFP associate with mitotic chromosomes

**Fig 1:**
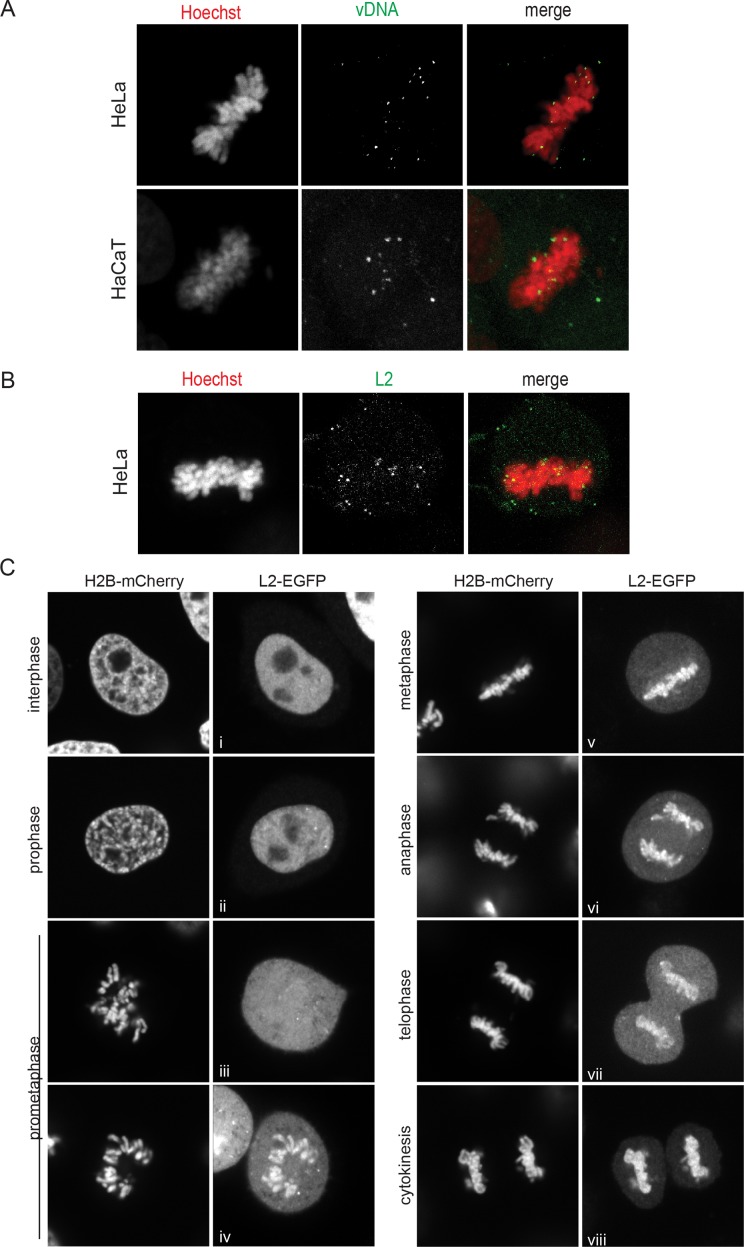
HPV16 vDNA and L2-EGFP associate with mitotic chromosomes. (A) HeLa cells and HaCaT cells were infected with EdU-HPV16, fixed at 20 h p.i., and stained for host DNA and vDNA using Hoechst and EdU-click chemistry, respectively. Cells were analyzed by CLSM. Shown is a representative confocal section of a metaphase cell with the host DNA (left, red), vDNA (center, green), and merge (right). (B) HeLa cells were infected with HPV16 PsV, fixed at 20 h p.i., and stained for incoming L2 protein using K1L2 and host DNA with Hoechst. Shown is a representative confocal section of a metaphase cell as above. (C) HeLa H2B-mCherry cells were transiently transfected with a HPV16 L2-EGFP expression plasmid, and synchronized by a single thymidine block. High magnification images of fixed cells were acquired using a spinning disc microscope. Depicted are representative images of the subcellular localization of L2-EGFP during the different cell cycle phases as determined by the histone marker.

Our previous work using infection of HPV16 PsVs containing EdU-labeled vDNA (EdU-HPV16) showed that incoming vDNA associates increasingly with condensed, mitotic chromosomes until metaphase, whereupon the degree of association remained constant [5]. As a reference for the metaphase localization of EdU-labeled DNA in EdU-HPV16 infected HeLa and HaCaT cells, please refer to Fig 1A. Live-cell imaging of L2-EGFP-expressing cells showed that HPV16 L2-EGFP localizes dynamically within the cell during cell division [5]. L2 is nuclear during interphase, disperses throughout the cell upon NEBD, and associates with chromosomes in metaphase. Thus, we proposed that L2 tethers the viral genome to mitotic chromosomes upon NEBD.

While live-cell imaging provides an excellent dynamic resolution of events, its primary caveat is a low spatial resolution. For a higher spatial resolution, the localization of HPV16 L2-EGFP was examined in transfected HeLa cells using laser scanning confocal microscopy (Fig 1C). After nuclear localization in interphase and prophase (Fig 1C, images i, ii), HPV16 L2-EGFP first associated with chromosomes during prometaphase (Fig 1C, image iii), and remained associated throughout metaphase, anaphase and telophase (Fig 1C, images v-vii). L2-EGFP association also occurred in HaCaT keratinocytes, or when L2 was tagged with a short HA epitope instead of EGFP suggesting that recruitment to mitotic chromatin is cell-type- and EGFP tag-independent (S1A and S1B Fig). Similar to the vDNA, L2 from incoming HPV16 PsV localized to metaphase chromosomes indicating that the L2-EGFP association with mitotic chromosomes in transfected cells phenocopies the recruitment during infection (Fig 1B).

The chromosomal recruitment of L2 during a specific stage of mitosis suggests that L2 may interact with a cellular protein that itself is specifically recruited to prometaphase chromosomes, rather than a direct L2 engagement of histones or cellular DNA. Based on the low spatially resolved live-cell imaging data, we had previously concluded that HPV16 L2 is recruited to mitotic chromosomes during metaphase [5]. However, the high-resolution microscopy data clearly indicates that recruitment of L2 occurs specifically during prometaphase, and does not require establishment of the metaphase plate. Furthermore, the accompanying paper by Calton et al. demonstrates that during HPV16 PsV infection of synchronized cells, EdU-labeled L2/vDNA complex begins to associate with mitotic chromosomes specifically during prometaphase, and essentially all of the incoming L2/vDNA is chromosomally associated by metaphase, confirming our previous observations [5, 47]. Importantly, this suggests that transfected L2-EGFP associates with mitotic chromosomes using the same mechanisms as incoming vDNA-associated L2 during PsV infection.

### L2-EGFP associates with chromosomes of nocodazole-arrested prometaphase cells

To verify recruitment of L2 to prometaphase chromosomes, L2-EGFP expressing cell populations were enriched in prometaphase using nocodazole. Nocodazole is a microtubule-depolymerizing drug that prevents the formation of the mitotic spindle, and therefore congression of mitotic chromosomes to the metaphase plate [48]. Thus, HeLa cells that stably expressed the histone marker H2B-mCherry (HeLa H2B-mCherry) were transfected with an L2-EGFP expression plasmid, and subsequently treated overnight with nocodazole prior to fixation and microscopy (S2A Fig). While only a small fraction of cells was in mitosis in the absence of nocodazole (S2B Fig, left, arrowheads), cells were efficiently enriched in prometaphase in the presence of the drug (S2B Fig, right). In these prometaphase-arrested cells, L2-EGFP associated with the chromosomes, whereas EGFP alone failed to accumulate on chromatin (S2C and S2D Fig). These results further validate that L2 is recruited to mitotic chromatin during prometaphase. Moreover, enriching cells in prometaphase allows for a steady-state analysis of mitotic chromosomal association of L2-EGFP in statistically significant numbers of cells.

### A conserved, central region in HPV16 L2 mediates association with mitotic chromosomes

**Fig 2:**
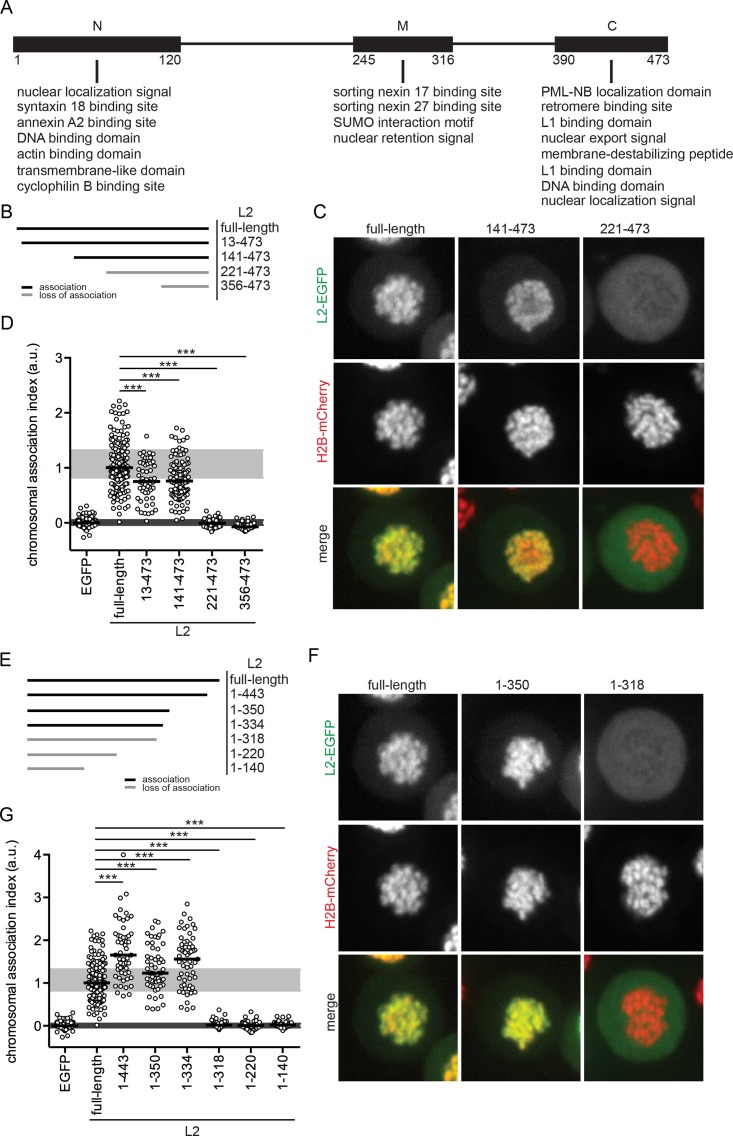
Chromosomal association of N-terminal or C-terminal deletion mutants of HPV16 L2. (A) Schematic illustration of L2 that can be divided into an N-terminal (N), middle (M), and C-terminal (C) part according to the clusters of described functional domains for PV L2. Numbers refer to the amino acid positions in HPV16 L2. (B) Overview of the N-terminally truncated HPV16 L2-EGFP mutants that were analyzed for chromosomal association during mitosis (numbers indicate the amino acid residues). The chromosomal association assay was performed upon transfection with L2-EGFP expression plasmids of the indicated PVs. Images were acquired using a spinning disc microscope with 40x magnification, and analyzed computationally. (C) Shown are single confocal slices of full-length and selected truncated HPV16 L2-EGFP (upper row, green), H2B-mCherry (center row, red), and merges (lower row) for representative cells (the remaining constructs are shown in S3 Fig). (D) Chromosomal association of L2 was quantified by a semi-automated image analysis algorithm of at least 50 cells. The chromosomal association was assessed by the chromosomal association index (CAI), i.e. the ratio of mean EGFP fluorescence intensity (intensity per area) of chromosomally associated L2-EGFP over cytoplasmic L2-EGFP normalized by subtracting the median ratio for EGFP. The CAI is depicted as dot plot with solid lines indicating the median, and grey areas marking the middle 50% range of data points for HPV16 L2-EGFP (light grey) and EGFP (dark grey). Statistical significances (two-tailed Student’s t-test) relative to full-length HPV16 L2-EGFP were assessed (***P < 0.001). (E) Overview of the C-terminal deletion mutants of HPV16 L2-EGFP that were examined in the chromosomal association assay. (F) Single confocal slices of full-length and selected C-terminally shortened HPV16 L2-EGFP (upper row, green), H2B-mCherry (center row, red) and merges (lower row) are shown for representative cells (the remaining constructs are shown in S3 Fig). (G) The chromosomal association indices for C-terminal deletion mutants are displayed as in (D).

L2 is multifunctional, facilitating several steps of the viral life cycle in concert with certain host factors: extracellular structural modifications of the capsid prior to infectious cell entry (furin cleavage, cyclophilin B binding domain, annexin A2 binding [19, 25, 49]), vesicular trafficking and endosome escape (cyclophilin B, sorting nexin 17, sorting nexin 27, retromer, syntaxin 18 binding domains, TM domain, membrane-destabilizing peptide, NRS [25, 26, 37–40, 50–53]), cytosolic transport (dynein, beta-actin [43, 44, 54]), and particle assembly (NLSs, nuclear export signal, DNA-binding, L1-binding, PML nuclear body-localization, SUMO-interaction domain [28, 30, 31, 33, 45]). Thereby, most of the known functional domains cluster towards the termini of the PV L2 protein, and only a few are located in the centre (Fig 2A, [29]).

To identify the potential chromosome-binding site(s) in HPV16 L2, chromosomal association of L2-EGFP truncation mutants was quantified in prometaphase-arrested cells. As an unbiased and quantitative measure, the chromosomal association index (CAI) was developed. Instead of using a threshold-based algorithm like for example colocalization analysis, the CAI uses non-arbitrary intensity based measurements. It is defined as mean fluorescence intensity ratio of chromosome-associated over cytoplasmic L2-EGFP. To correct for artificial background, these values were normalized to control EGFP-expressing cells. First, HPV16 L2 was sequentially truncated from the N-terminus (Fig 2B). L2(13–473)-EGFP and L2(141–473)-EGFP, lacking all the N-terminal functional domains, visually associated with mitotic chromosomes similar to full-length L2-EGFP (Fig 2C, S3A Fig). The CAI analysis of transfected cells revealed that chromosomal association of L2(13–473)-EGFP and L2(141–473)-EGFP was decreased by a small but significant amount (Fig 2D), which suggests that N-terminal residues up to amino acid (aa) 140 may partially stabilize chromosomal association. Remarkably, chromosomal association is completely abolished upon deletion of further N-terminal residues with unknown function: L2(221–473)-EGFP and L2(356–473)-EGFP were excluded from the chromosomes exhibiting CAIs similar to the non-associating EGFP (Fig 2C and 2D). Thus, residues in between aa 141 and 220 are crucial for mitotic chromosome binding, whereas the functionally described N-terminal domains are dispensable.

Next, L2 C-terminal deletions were tested (Fig 2E). L2(1–443)-EGFP lacking the C-terminal DNA-binding domain, NLS, nuclear export signal, dynein-interacting domain, and membrane-destabilizing peptide [30, 31, 33, 40, 43], and L2(1–350)-EGFP additionally lacking the PML localization, L1 and retromer interaction domains [28, 32, 34], clearly associated with mitotic chromosomes (Fig 2F and 2G; S3B Fig), suggesting that the C-terminal functional domains are dispensable for chromatin association. Interestingly, sequential C-terminal truncations up to aa 334 as in L2(1–443)-EGFP, L2(1–350)-EGFP, and L2(1–334)-EGFP resulted in increased association, reflected by median CAIs up to 60% higher than that of full-length L2-EGFP (Fig 2F and 2G, S3B Fig). Thus, the C-terminal functional domains are partially inhibitory for chromosomal association. This may indicate that L2 interactions with, for example, retromer or dynein, need to be reversed for efficient chromosomal association during initial infection and subcellular trafficking of L2/vDNA. Notably, chromosomal association was completely lost in truncations lacking 155 or more amino acids from the C-terminus such as in L2(1–318)-EGFP, L2(1–220)-EGFP and L2(1–140)-EGFP (Fig 2F and 2G, S3B Fig), suggesting a crucial role for aa 319 to 334 in mitotic chromosomal association of L2. In summary, the C-terminal functional domains are dispensable for chromosomal association similar to the N-terminal domains.

**Fig 3:**
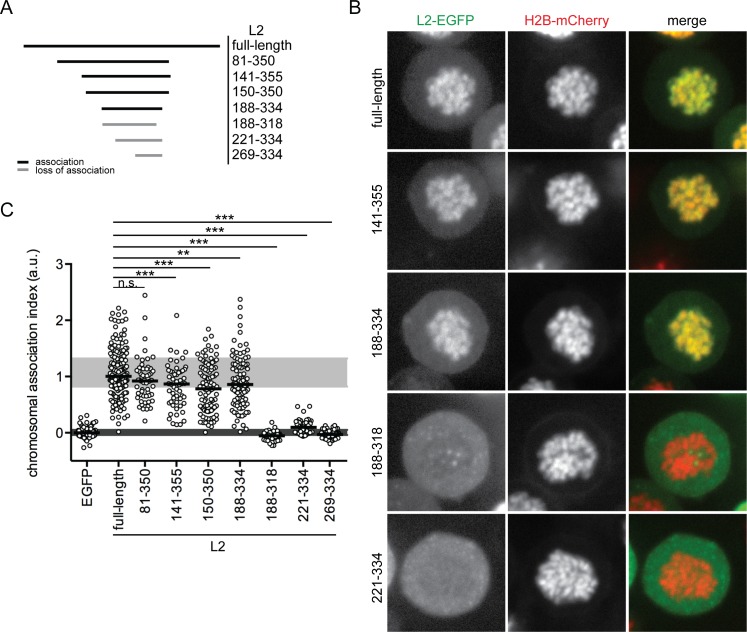
Chromosomal association of bilateral truncations of HPV16 L2. (A) Overview of the bilaterally truncated HPV16 L2-EGFP that were assayed for chromosomal association (numbers indicate the amino acid residues). (B) Images of full-length and selected fragments of HPV16 L2-EGFP (left, green), H2B-mCherry (center, red), and merges (right, merge) are depicted for representative cells (the remaining constructs are shown in S3 Fig). (C) The chromosomal association indices of the N- and C-terminally truncated versions of L2-EGFP are plotted as in Fig 2D. Statistical significances (two-tailed Student’s t-test) relative to full-length HPV16 L2-EGFP were determined (n.s. = not significant, **P < 0.01, ***P < 0.001).

To further exclude that the N- and C-terminal domains compensate each other for mitotic chromosome recruitment, bilaterally truncated L2-EGFP mutants were tested (Fig 3A). Since, for example, L2(141–355)-EGFP was fully capable to bind to mitotic chromosomes, it is unlikely that compensation occurs (Fig 3B and 3C, S3C Fig). The minimal peptide that binds to chromosomes was L2(188–334)-EGFP (Fig 3B and 3C). Further truncation of this central L2 fragment from either end abrogated chromosomal association, as evidenced by L2(188–318)-EGFP and L2(221–334)-EGFP (Fig 3B and 3C). The requirement for residues directly upstream of aa 221, and residues between aa 319 and 334 correlated with the abolished chromosome-binding of L2(221–473)-EGFP and L2(1–318)-EGFP (Figs 2, 3 and S3). Since the central region of 147 amino acids (aa 188 to 334) is necessary and sufficient to mediate association of HPV16 L2 with mitotic chromosomes, we designate it the chromosome-binding region (CBR).

### Association of L2 with mitotic chromosomes is conserved among PVs

**Fig 4:**
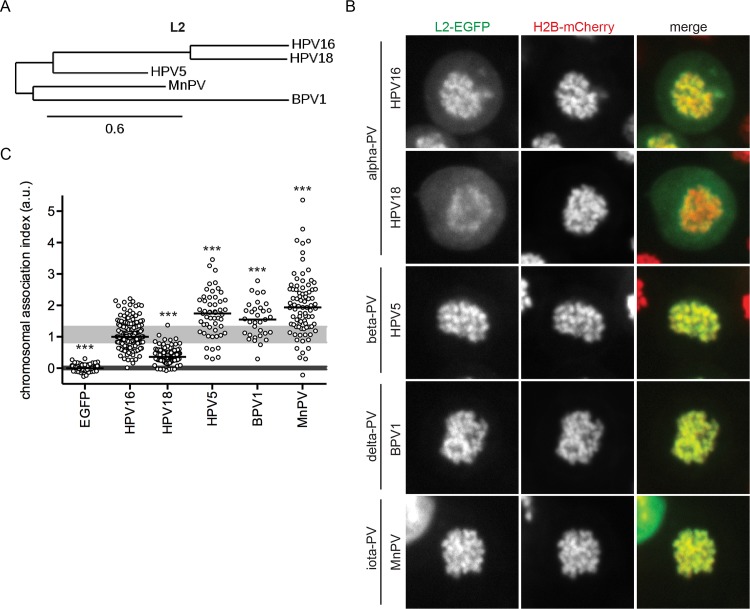
Association of L2 with mitotic chromosomes is conserved among PVs. (A) The phylogram illustrates the evolutionary relationships between the mucosal high-risk alpha-PVs HPV16 and HPV18, the cutaneous beta-PV HPV5, and the animal delta- and iota-PV BPV1 and MnPV, respectively, based on their L2 amino acid sequences. The branch length represents the amount of genetic changes (scale bar: 0.6 substitutions/site). (B) Images of L2-EGFP (left, green), H2B-mCherry (center, red), and merges (right) are shown for representative cells. (C) The chromosomal association indices for N-terminal deletion mutants are depicted as in Fig 2D. As an exception, only 33 cells were analyzed for BPV1 L2.

To assess the functional conservation of L2-chromosome interactions among PVs of different genera, L2 association assays were carried out for HPV18 (another mucosal high-risk alpha-PV), HPV5 (cutaneous low-risk beta-PV), BPV1 (bovine delta-PV), and MnPV *(Mastomys natalensis* iota-PV). The L2 sequences of HPV16 and HPV18 are evolutionary most closely related, followed by HPV5, MnPV and BPV1 (Fig 4A). L2-EGFP of all tested PVs associated with prometaphase chromosomes, albeit to varying extents (Fig 4B and 4C). L2-EGFP of HPV5, BPV1 and MnPV were distributed evenly over the mitotic chromosomes similar to HPV16 (Fig 4B). In contrast, HPV18 L2-EGFP localized to the peripheral rim of chromosomes (Fig 4B), indicating that HPV18 L2 may be recruited in a different mode or to a different extent. Quantitatively, HPV18 L2-EGFP associated considerably less with chromosomes than HPV16 L2-EGFP (Fig 4B and 4C). In contrast, L2-EGFP of HPV5, and the animal PVs had substantially higher CAIs than HPV16 (Fig 4B and 4C). Hence, certain L2s may engage a common cellular recruiting factor with different affinities, or engage different cellular factors for recruitment to mitotic chromatin.

**Fig 5:**
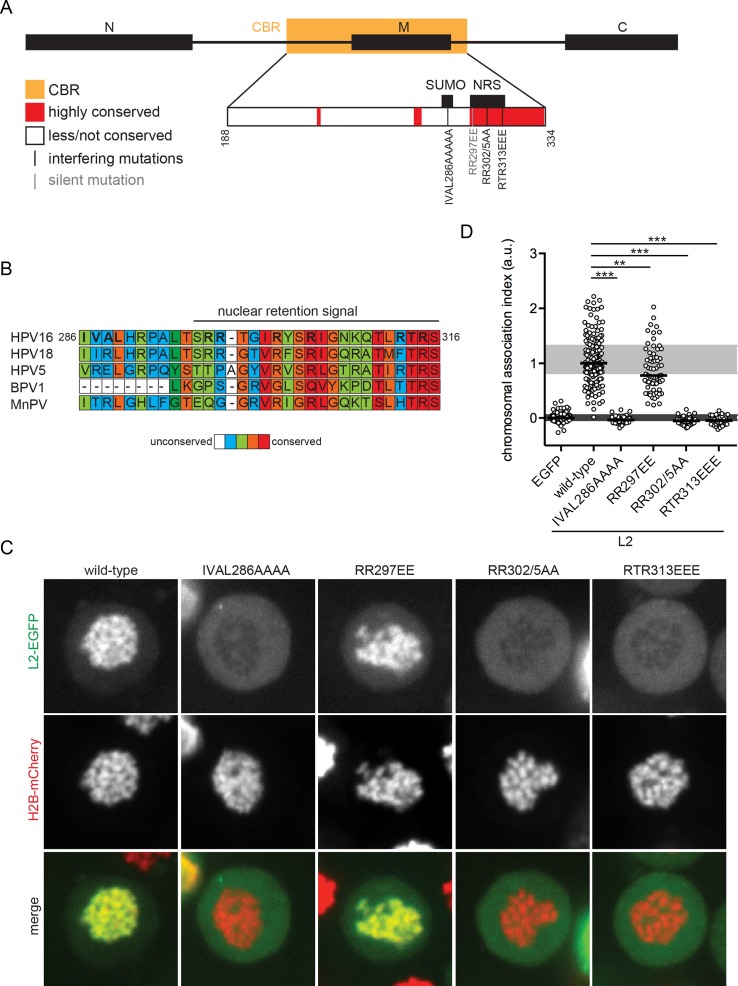
Chromosomal association of point mutants of HPV16 L2. (A) Full-length L2 is depicted with N-terminal, middle and C-terminal functional domains. The minimal chromosomal binding region (CBR) is highlighted in orange. Within the CBR (insert) the nuclear retention signal and the SUMO interaction motif are indicated at their relative location with the CBR. Conserved regions within the CBR are depicted in red, whereas regions with lower conservation are white. Single conserved residues are highlighted. The sites of the point mutations in the CBR are color-coded according to their impact on chromosomal association (black = interfering; grey = silent). (B) The alignment of the amino acid (aa) sequences of the NRS and upstream residues of HPV16, 18, 5, BPV1 and MnPV L2 are written in the single-letter code. The aa residue numbers, and mutated residues (bold font) are denoted for HPV16 L2. The conservation between the indicated L2 aa sequences was scored by PRALINE [78], and obtained scores were grouped into five categories: no (white), low (blue), intermediate-low (green), intermediate-high (orange) and high (red) conservation. (C), (D) The aa substitutions IVAL286AAAA, RR297EE, RR302/5AA and RTR313EEE in HPV16 L2-EGFP were analyzed for chromosomal association during mitosis as in Fig 2C and 2D. (C) Images of wild-type and mutant HPV16 L2-EGFP (upper row, green), H2B-mCherry (center row, red), and merges (lower row) are shown for representative cells. (D) The chromosomal association indices are depicted in a dot plot as in Fig 2D. Statistical significances (two-tailed Student’s t-test) relative to wild-type HPV16 L2-EGFP were assessed (**P < 0.01, ***P < 0.001).

Overall, the tested PV L2s were all recruited to mitotic chromatin suggesting a similar tethering strategy. A comparison of the CBR of the analyzed PV types revealed that it contains two parts, a variable N-terminal region from aa 188 to 301 that contains eight conserved residues (aa 251 to 258), and a highly conserved C-terminal region from aa 302 to 334, overlapping with the previously defined NRS (Fig 5A). The different degrees and patterns of association are likely the result of the variable regions within the CBR. To further elucidate the role of the CBR, we focused on conserved residues within L2 assuming that conservation likely correlates with functional importance.

### Point mutations in HPV16 L2 abolish association with mitotic chromosomes

Directly upstream of, or within the highly conserved NRS, the CBR contains residues of several previously described mutations (Fig 5B). Upstream of the NRS, the SUMO-interacting motif IVAL286 is required for efficient HPV infection and important for intranuclear targeting of the incoming vDNA to PML-NBs [45]. Glutamic acid substitutions of critical arginine residues within the NRS, as e.g. RR297 and RTR313, reduce the fraction of exogenously expressed L2 in the nucleus despite the presence of both NLSs [31]. Further alanine-substitution studies of residues RR302/5 revealed that the NRS is essential for infection [26, 42]. Interestingly, these studies from the Sapp laboratory variably attribute the function to different entry steps, i.e. traversal of the TGN or nuclear import [26, 42].

To directly test whether these residues would be crucial for recruitment of L2 to mitotic chromosomes, respective mutations were introduced into HPV16 L2-EGFP by site-directed mutagenesis, and tested for chromosomal association (Fig 5B). Of those mutants, only L2(RR297EE)-EGFP associated with mitotic chromosomes although to a lesser extent compared to wild-type L2-EGFP (Fig 5C and 5D). In contrast, L2(IVAL286AAAA)-EGFP, L2(RR302/5AA)-EGFP, and L2(RTR313EEE)-EGFP are unable to bind mitotic chromatin (Fig 5C and 5D). For ease of experimentation, HeLa H2B-mCherry cells were used for most of our assays. For confirmation, association was also tested for L2(IVAL286AAAA)-EGFP and L2(RTR313EEE)-EGFP in HaCaT keratinocytes. Similar to HeLa cells, association was abrogated for these mutants in HaCaT cells (S1B and S1C Fig). Thus, L2 residues IVAL286, RR302/5, and RTR313 are involved in L2 association with mitotic chromosomes, and likely play a role in viral genome tethering to mitotic chromatin for nuclear entry.

### Mutant PsVs are impaired in nuclear delivery of vDNA and L2

To verify the role of these mutations under more physiological conditions, HPV16 PsVs harbouring L2(RTR313EEE) and L2(IVAL286AAAA) were tested in infection studies. As a structural component of the virus capsid, L2 is involved in virion assembly as well as virus entry (reviewed in [29]). Since virions containing L2(IVAL286AAAA) are known to be assembly-competent [45], we tested whether mutant HPV16 PsVs harbouring L2(RTR313EEE) would also assemble properly. Morphologic analysis of HPV16-L2(RTR313EEE) PsVs by negative stain EM revealed no detectable differences between wild-type and mutant HPV16 particles (S4A Fig). In addition, wildtype and mutant L2 proteins, and the EGFP-reporter pseudogenome were incorporated into virus particles with comparable efficiency as shown by densitometric analysis of Coomassie-stained SDS-PAGE gels of PsVs (S4B Fig). Thus, the mutant HPV16-PsVs assemble without considerable defects.

**Fig 6:**
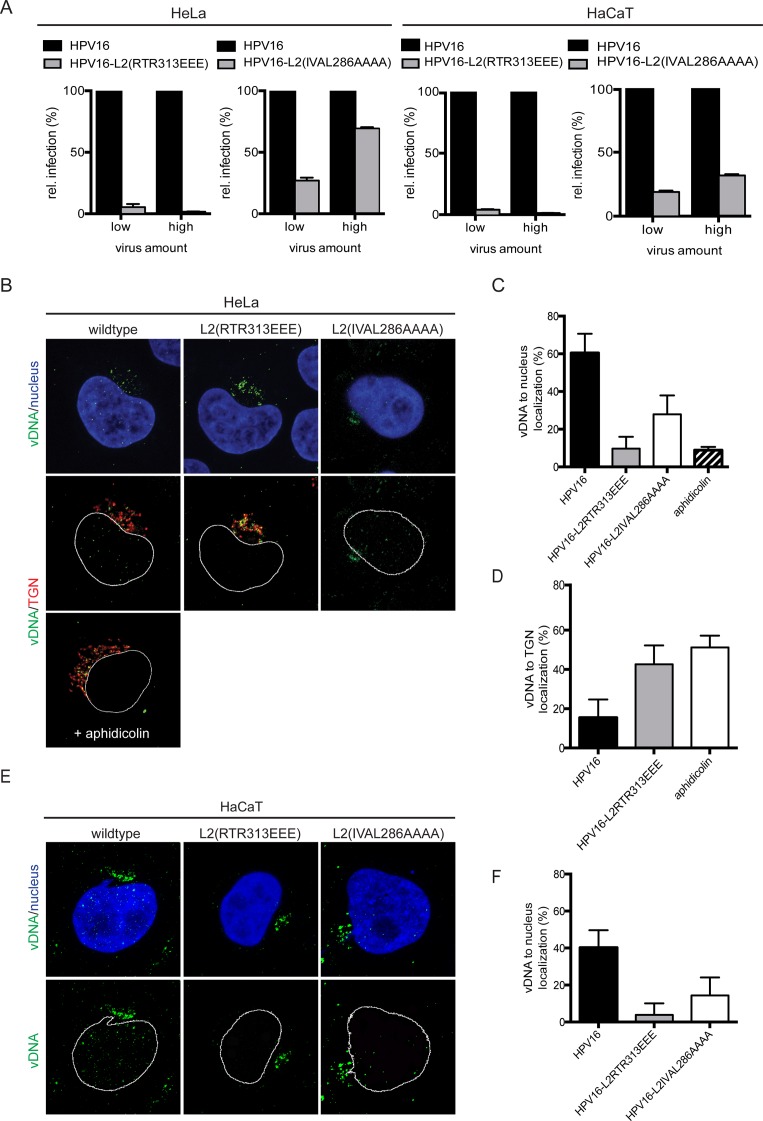
Mutant HPV16 PsVs are impaired in nuclear delivery of vDNA. (A) HeLa cells and HaCaT cells were infected with low and high amounts of HPV16, HPV16-L2(RTR313EEE) and HPV16-L2(IVAL286AAAA) PsVs carrying a GFP reporter plasmid, fixed at 48 h p.i., and analyzed by flow cytometry. Depicted is the fraction of GFP-positive (i.e. infected) cells in percent ± SD. (B) Subcellular localization of incoming vDNA upon infection with HPV16, HPV16-L2(RTR313EEE) and HPV16-L2(IVAL286AAAA) PsVs carrying EdU-labeled vDNA in the absence or presence of 15 μM aphidicolin was analyzed in HeLa cells at 20 h p.i. by CLSM. Depicted are maximum intensity projections of three medial confocal slices of vDNA detected by EdU-click chemistry (green), host nucleus stained with Hoechst (blue), and the trans-Golgi network immunofluorescently stained for p230 (TGN, red). Shown are merge images of vDNA and nucleus (upper row), and vDNA and TGN in the absence and presence of aphidicolin (middle an lower row, respectively). (C) Quantification of intensity-based colocalization (in percent ± SD) of vDNA from EdU-HPV16, EdU-HPV16-L2(RTR313EEE) and EdU-HPV16-L2(IVAL286AAAA) with the host nucleus. (D) Quantification of intensity-based colocalization (in percent ± SD) of vDNA from EdU-HPV16, and EdU-HPV16-L2(RTR313EEE) with the TGN upon infection in the absence or presence of aphidicolin. (E) Subcellular localization of incoming vDNA upon infection with HPV16, HPV16-L2(RTR313EEE) and HPV16-L2(IVAL286AAAA) PsVs carrying EdU-labeled vDNA was analyzed in HaCaT cells at 20 h p.i. by CLSM. Depicted are maximum intensity projections of three medial confocal slices of vDNA detected by EdU-click chemistry (green) and host nucleus stained with Hoechst (blue). Shown are merge images of vDNA and nucleus (top panel), and vDNA with the nuclear outline (lower panel). (F) Quantification of intensity-based colocalization was performed as in (C).

Next, we assessed whether incorporation of mutant L2 into virions would affect infectious entry. For this, HeLa or HaCaT cells were infected with HPV16, HPV16-L2(RTR313EEE), and HPV16-L2(IVAL286AAAA) at low and high multiplicities. In line with published results [45], incorporation of L2(IVAL286AAAA) severely reduced infectivity of the PsVs in both HeLa and HaCaT cells (Fig 6A), however, some infectivity of this mutant was retained at the higher MOI (Fig 6A). HPV16-L2(RTR313EEE) PsVs were non-infectious in both cell types (Fig 6A and 6B). While both motifs are important for infectivity, they differ in their ability to perturb virus entry and possibly virus tethering. Interestingly, chromosomal association was similarly abrogated for both mutants, suggesting that this assay is extremely sensitive.

**Fig 7:**
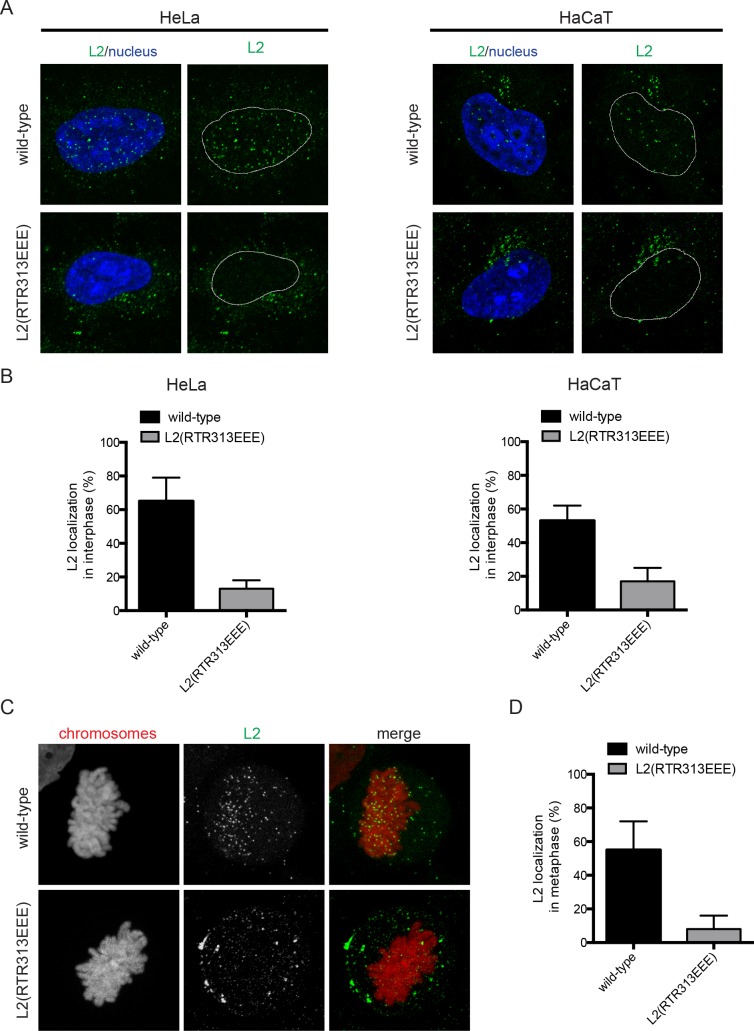
Mutant HPV16 PsVs are impaired in nuclear delivery of L2(RTR313EEE). (A) HeLa or HaCaT cells were infected with HPV16 wild-type or HPV16-L2(RTR313EEE) PsVs. At 20 h p.i. cells were fix with 4% PFA and stained for the localization of incoming L2 protein with K1L2 antibody and with Hoechst for the host DNA. Depicted are merge images of L2 and nucleus (left panel) and L2 signal with nuclear outline (right panel) for HPV16 wild-type (upper row) and HPV16-L2(RTR313EEE) (lower row). (B) Quantification of intensity-based colocalization (in percent ± SD) of L2 protein from HPV16 wild-type and HPV16-L2(RTR313EEE) with the host nucleus in HeLa and HaCaT cells. (C) HeLa H2B-mCherry were infected with HPV16 wild-type or HPV16-L2(RTR313EEE) PsVs. At 20 h p.i. cells were fixed, stained and analyzed as in (A). (D) Quantification of intensity-based colocalization (in percent ± SD) of L2 protein from HPV16 wild-type and HPV16-L2(RTR313EEE) with the mitotic host chomosomes in HeLa H2B-mCherry cells.

Next, the subcellular localization of vDNA upon infection was analyzed to test whether mutant PsVs failed to deliver vDNA to the nucleus. Intensity-based colocalization analysis showed that at 20 hours post infection (h p.i.) EdU-HPV16 had successfully delivered 60%±10% and 40±12% of vDNA to HeLa and HaCaT nuclei, respectively (Fig 6B, 6C, 6E and 6F). In contrast, intranuclear localization of vDNA was reduced or absent, when cells were infected with EdU-HPV16-L2(IVAL286AAAA) or EdU-HPV16-L2(RTR313EEE) PsVs, respectively (Fig 6B, 6C, 6E and 6F). Consistent with a defect in viral genome tethering, both mutants exhibited defects to direct vDNA to nuclei of infected host cells. Instead, the vDNA of mutant virions accumulated in perinuclear clusters, reminiscent of TGN localization. Similar to the infection studies, the L2(RTR313EEE) mutant PsV exhibited stronger defects than L2(IVAL286AAAA). To verify these results, the localization of L2 was analyzed by antibody staining. Similar to the vDNA, L2 failed to localize to host cell nuclei after HPV16-L2(RTR313EEE) infection (Fig 7A and 7B).

The perinuclear accumulation of vDNA suggested that the mutant L2 proteins were able to direct pseudogenomes to the TGN. Accordingly, vDNA of EdU-HPV16 and EdU-HPV16-L2(RTR313EEE) were detectable in the TGN (Fig 6B and 6D). In fact, the mutation caused increased accumulation of vDNA at the TGN (Fig 6B and 6D), similar to the “Golgi retention” phenotype previously observed with EdU-labeled RR302/5AA mutant PsV [26]. As a control, cells were treated with aphidicolin, which arrests cells in interphase, and inhibits infection by blocking nuclear entry of wild-type HPV16 [5, 6]. As expected, vDNA of EdU-HPV16 was prevented from entering the nucleus under these conditions and accumulated in the TGN (Fig 6B and 6D).

**Fig 8:**
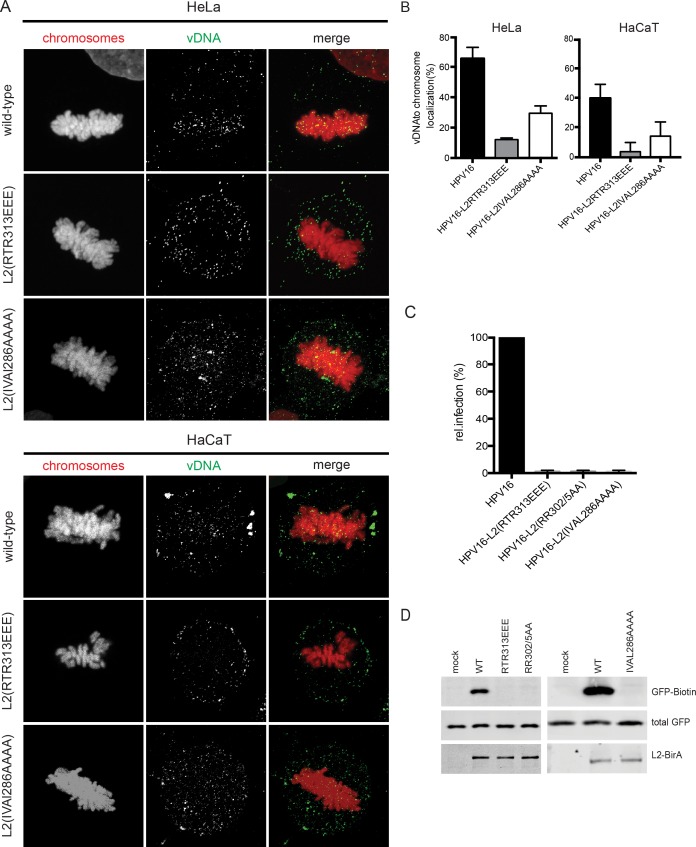
Mutant PsVs are defective in mitotic chromosome association of vDNA and membrane penetration of L2 C-terminus. (A), (B) Subcellular localization of incoming vDNA upon infection with EdU-HPV16, EdU-HPV16-L2(RTR313EEE) and EdU-HPV16-L2(IVAL286AAAA) in mitotic HeLa H2B-mCherry and HaCaT cells. Cellular chromatin in HaCaT cells was visualized by Hoechst staining. (A) Depicted are maximum intensity projections of confocal slices of host chromatin (left panel, red) and vDNA (center panel, green), and merge images (right panel). (B) Quantification of intensity-based colocalization (in percent ± SD) of vDNA from EdU-HPV16 and EdU-HPV16-L2(RTR313EEE) with the host chromatin. (C) Infection and translocation in HaCaT GFP-BAP cells infected with HPV16 L2, L2(RTR313EEE), L2(RR302/05AA), or L2(IVAL286AAAA)-BirA PsV at equal multiplicities of infection. GFP-biotin, total GFP, and intracellular L2-BirA were visualized by Western blot and immunostaining with neutravidin, anti-GFP, and anti-L2 (K4) respectively, as described in [47]. (D) Infection values were determined by luciferase measurements and represent mean relative infection values compared to wt L2-BirA infected cells and normalized to GAPDH levels.

Thus, the mutants were able to traffic vDNA to the TGN but failed to deliver vDNA to the nucleus, instead resulting in vDNA accumulation at the TGN, presumably due to a defect in tethering to mitotic chromosomes for nuclear import. In support, association of vDNA with metaphase chromosomes of mitotic HeLa and HaCaT cells was substantially impaired for EdU-HPV16-L2(IVAL286AAAA) and virtually absent for EdU-HPV16-L2(RTR313EEE) (Fig 8A and 8B). Similarly, L2(RTR313EEE) did not localize to metaphase chromosomes after infection of HeLa cells, whereas wild-type L2 accumulated on mitotic chromatin (Fig 7C and 7D).

The Golgi retention phenotype and defective nuclear entry shared between the defective CBR mutants L2(IVAL286AAAA), L2(RTR313AAA), and L2(RR302/5AA) (the latter reported in [26]) prompted us to determine whether these mutants could translocate across limiting membranes during infection. The accompanying report by Calton and colleagues [47] describes a novel system for measuring L2 membrane penetration. In brief, the assay utilizes PsVs that encapsidate L2, which is C-terminally fused to the BirA biotin ligase from *E*. *coli*. BirA catalyzes the covalent addition of free biotin to a specific 15-residue biotin acceptor peptide (BAP) substrate [55]. L2-BirA PsVs are used to infect a HaCaT cell clone that stably expresses a cytosolic GFP-BAP fusion [47]. Only upon translocation of the C-terminus of L2-BirA across limiting membranes can the BirA moiety encounter and biotinylate the GFP-BAP substrate. Thus, the degree of biotinylation of GFP-BAP is a proxy for the efficiency of translocation of the L2 C-terminus across the limiting membrane of vesicular compartments [47]. Using this system, Calton et al. observed that translocation is dependent upon the cell cycle, with a strict requirement for entry into mitosis. Furthermore the timing of translocation in synchronized cells was coincident with or just subsequent to mitotic onset [47].

Using the L2-BirA system, Calton et al. show that the L2(RR302/5AA) mutant, which is defective for chromatin binding (Fig 5), was impaired for translocation of the L2 C-terminus across the limiting membrane. Thus, we tested whether L2(RTR313EEE) and L2(IVAL286AAAA) would also be unable to penetrate. HaCaT GFP-BAP cells were infected with HPV16 L2-BirA PsVs containing L2(RTR313EEE), L2(IVAL286AAAA), or, as control, L2(RR302/5AA). In comparison to the wild-type particles, the mutant L2-BirA PsVs were noninfectious and unable biotinylate GFP-BAP, indicating that the L2 region important for binding mitotic chromosomes is also required for translocation of the L2 C-terminus into the cytoplasm (Fig 8C and 8D).

Overall, this study further validates the model of PV nuclear entry, in which L2 tethers the viral genome to mitotic chromosomes upon NEBD in order to direct it to the nascent nuclei during cell division. Moreover, a central region in the HPV16 minor capsid protein L2 from aa 188 to 334 was identified as sufficient and required for association of L2 with mitotic chromosomes. In particular, we characterized the chromosome-binding deficient mutant L2(RTR313EEE) that rendered mutant PsVs non-infectious. HPV16-L2(RTR313EEE) PsVs were unable to deliver the vDNA to the nucleus and were impaired for C-terminal translocation across the membranes of the vesicular compartment. Taken together, these results supported a new model in which L2 chromatin binding and translocation are interdependent processes. Finally, we provide evidence that *Papillomaviridae* across different genera share a common L2-mediated nuclear entry strategy by tethering vDNA to mitotic chromosomes.

## Discussion

Our recent work demonstrated that nuclear delivery of HPV16 genomes requires NEBD during mitosis, and it provided first evidence for the existence of a tethering mechanism of the viral genome to mitotic chromatin by the minor capsid protein L2 [5]. In this study, we demonstrated that a conserved, central CBR encompassing amino acid residues 188 to 334 of HPV16 L2 mediates recruitment to mitotic chromosomes specifically during prometaphase. By tethering vDNA to mitotic chromosomes, L2 facilitates nuclear delivery of incoming vDNA and infection of both daughter cells. Our data also show that conserved residues within the CBR are also critical for membrane penetration of the L2 C-terminus, suggesting that these two processes are functionally linked.

To ensure inclusion into the nascent daughter nuclei after cell division, the vDNA and L2 interact with host mitotic chromatin. This interaction is consistent with L2 acting as a viral tethering factor. A tethering function implies that one part of the protein holds on to vDNA, whereas another part interacts with host chromatin [56]. In PV L2, both the N- and C-terminal polybasic regions of L2 are capable of binding to DNA in vitro [33, 57, 58] and these linear DNA-binding domains may bind the vDNA during assembly of the virions [33, 57–59]. Prior work with purified full length and truncated BPV1 L2 suggests that in particular the C-terminal domain mediates direct binding to DNA [58]. Furthermore, for incoming virions, the N-terminal DNA-binding domain of L2 is cleaved off by furin to mediate localization of L2/vDNA to the TGN [19] [24]. Thus, the vDNA is likely attached to the L2 C-terminal DNA-binding domain upon virus entry and it is unlikely that any of these terminal DNA-binding domains would be available for host chromatin binding. Moreover, our results demonstrate that mitotic chromosomal association of HPV16 L2 is independent of both N-terminal and C-terminal DNA-binding domains, and that the central CBR in HPV16 L2 was sufficient and necessary for chromosomal association. Hence, the use of two different sites in L2 would clearly allow simultaneous binding to mitotic chromatin and the vDNA, the prerequisite for a tethering factor.

Two distant sites within the CBR, L2(189–220) and L2(319–334), are alone insufficient but indispensable for chromosomal association. Both sites may be necessary to form an interface that binds host mitotic chromosomes upon folding. Since L2 is for the most part, predicted to be an intrinsically disordered protein (DisEMBL, [60]), it is likely that these sites act as linear motifs independent of protein secondary and tertiary structures. In support, computational analysis predicted that both sites contain disordered loops with high mobility, which may fold upon binding to the corresponding cellular partner. Alternatively, L2 may function as a scaffold, in which each of these sites interacts with a different cellular chromosomal target, thereby stabilizing the association with chromatin. However, a simple stabilization appears less likely, as deletion of both L2 sites abrogates mitotic chromatin recruitment. Nevertheless, the potential for multivalent interactions to tether vDNA to chromatin is not without precedent: EBNA1 and LANA1 of Epstein Barr virus and Kaposi’s sarcoma associated herpesvirus, respectively, both use two N- and C-terminal chromosome-binding sites to tether the viral episome to chromosomes for maintenance (reviewed in [56]). The N-terminal chromosome-binding domain of LANA1 interacts with histones H2A-H2B at the nucleosomal surface and modulates the C-terminal interaction with chromosomally associated methyl-CpG-binding protein 2 [61, 62]. Interestingly, the C-terminal part (aa 302–334) of the central CBR of HPV16 L2 is highly conserved among PVs, and HPV16 L2 residues 312 to 322 exhibit high conservation with residues 6 to 16 of the N-terminal chromosome-binding domain of LANA1 [63]. While this might point to a putative interaction of HPV16 L2 with histones, such an interaction appears unlikely, as exogenously expressed L2 is recruited specifically and dynamically to mitotic chromosomes and does not interact with interphase and prophase chromatin. The prometaphase-dependent chromosomal association also argues against use of the L2 terminal DNA-binding domains to attach to host chromosomes, as these interactions are non-sequence specific and would be expected to occur throughout the cell cycle rather than at a specific stage of mitosis.

In order to engage a cellular factor for tethering during mitosis, the L2 CBR has to be cytosolic. Despite the growing knowledge of molecular players involved in PV intracellular trafficking, it still remains controversial when and how L2/vDNA penetrates and egresses from membrane-bound organelles. L2 contains an N-terminal TM domain and a C-terminal membrane-destabilizing peptide that are essential for PV infection [39, 40]. Precisely how these domains insert into the membrane remains unknown, but previous work suggests that L2 may adopt a type-I transmembrane topology during infection, whereby residues upstream of the TM domain are luminal and residues immediately downstream are cytosolic [41]. It should be noted, however, that the topology of the extreme C-terminus was never determined in this prior work.

The cytosolic exposure of residues downstream of the TM domain, including the central CBR, likely occurs within endosomal compartments because this portion of L2 contains motifs that are involved in endosomal sorting and trafficking of L2/vDNA to the TGN through interaction with cytosolic SNX17, SNX27, and the retromer [37, 38, 50]. Interestingly, the accompanying report from the Campos group provides indirect evidence that the extreme C-terminus of L2 together with the vDNA remains luminal within endosomes [47]. This may point to a topology with two membrane-spanning domains, a previously described N-terminally located domain, and a yet elusive C-terminally located domain. Thus, both termini of L2 would be luminal, and the CBR would be cytosolic in this model. The previously described C-terminal membrane destabilizing peptide could be a candidate for this additional membrane spanning domain, but this region does not have the biochemical characteristics of a TM domain [40]. Alternatively, it has been proposed that multiple L2 molecules may oligomerize within the membrane to form a pore, because the N-terminal TM domain of L2 can self-associate within biological membranes [39]. A pore-like structure built from the N-terminal TM domain might allow passage of the L2 C-terminus across the membrane without the assistance of another TM domain. Clearly, further work is necessary to determine the unique topology of L2 as it exists in this membrane-spanning state.

In any case, all of these models would suggest that the L2 CBR is accessible for interaction with cytosolic cellular interaction partners. Further, the CBR is located in between the SNX17 binding site at residues 254–257 and the retromer binding sites near the C-terminus. That both of these regions become exposed to the cytosol to engage their respective sorting molecules during infection strongly suggests that the CBR does as well.

Point mutational analyses of the highly conserved NRS, which is located within the identified CBR, further strengthened the potential role of this region in delivering vDNA to the nucleus by tethering the viral genome to mitotic chromosomes. The L2(RTR313EEE) mutation of mostly highly conserved residues has previously been reported to impair nuclear retention of exogenously expressed L2 [31]. Here, we show that this mutation abolished binding of L2 to mitotic chromatin, and rendered the assembly-competent HPV16 particles non-infectious. HPV16-L2(RTR313EEE) PsVs trafficked to the TGN but failed to reach the nucleus. Moreover, the mutant L2 was unable to tether vDNA to chromosomes during mitosis, which could explain the defect in nuclear entry. Our analysis of the previously described L2(RR302/5AA) mutation of conserved residues in the NRS in our chromosomal association assay revealed that this mutant was also unable to bind to mitotic chromosomes. Similar to our findings with HPV16-L2(RTR313EEE) PsVs, recent reports indicate that HPV16-L2(RR302/5AA) PsVs traffic to the TGN but fail to reach the nucleus [26, 42].

Interestingly, the L2(RTR313EEE) and the L2(RR302/5AA) mutations blocked translocation of the L2 C-terminus across the limiting membrane as well as L2 association with mitotic chromatin (Figs 5 and 8; [47]). Together these data strongly suggest that translocation and tethering to mitotic chromosomes are functionally linked. In support of this hypothesis, L2 translocation coincides with mitosis, and is inhibited if the cell cycle is arrested prior to M-phase [47]. Moreover, since the L2(RR302/5AA) and L2(RTR313EEE) mutations impair both the membrane translocation of the L2 C-terminus and the recruitment to mitotic chromatin[47], the interaction with a cellular tethering factor may additionally act as a translocation factor, functionally coupling L2 chromatin binding to membrane translocation of the L2/vDNA. If this is the case, the L2(RR302/5AA) and L2(RTR313EEE) mutations are likely located within the interface for an interaction with a cellular partner.

In addition to the mutations within the NRS, mutation of the less conserved SUMO interacting motif (IVAL286AAA) of HPV16 L2 adjacent to the NRS impaired mitotic chromatin association and membrane penetration. Interestingly, this mutation was only partially defective for nuclear import in infection studies in line with previous work [45], which may suggest that the conserved C-terminal part of the NRS contains the more crucial part of the interface for an interaction with a cellular partner. Alternatively, the mutations may have varying structural relevance by affecting the folding dynamics upon engagement of cellular targets at different sites of L2.

It has been suggested that L2(RR302/5AA) mutation may disrupt interaction of L2 with factors mediating plus end-directed transport along microtubules towards condensed chromosomes [42]. Our data show that recruitment of PV L2 to mitotic chromosomes can occur independently of microtubules, because L2 associates with prometaphase chromosomes in the presence of the microtubule-depolymerizing drug nocodazole. While this data does not exclude the possibility that kinesin-mediated transport occurs and contributes to the efficiency of chromosomal association during infection, L2 recruitment to mitotic chromatin is clearly efficient in the absence of kinesin-mediated microtubule transport.

Our work strongly indicates a common mechanism among PVs in which L2 tethers the vDNA to mitotic chromosomes for nuclear entry during cell division. For the first time, our results demonstrate that L2 proteins from three different host species (human, bovine, rodent) and four PV genera (alpha, beta, delta, iota) are recruited to mitotic chromosomes. Interestingly, differences in the pattern and extent of association between the different PV L2 proteins may indicate either differential affinities to the same cellular interaction partners or the engagement of different tethering factors. It is tempting to speculate about the latter, as this feature would not be unprecedented even among PVs. The PV E2 protein mediates genome maintenance during persistent PV infection in dividing cells by tethering the episomal genome to host chromatin (reviewed in [64]). While the N-terminus of the E2 protein of BPV1 interacts with bromodomain-containing protein 4 (Brd4) on mitotic chromosomes, HPV8 E2 binds pericentromeric regions, independently of Brd4, via its hinge region and C-terminal domain [65, 66]. If L2 proteins of different PVs would interact with different cellular factors, one would expect that the interaction would be primarily defined with less conserved residues of the CBR, as found in the N-terminal portion. If all PV L2 proteins would employ the same cellular chromosomal factors, the conserved residues of the L2 CBR may be responsible for the principle interaction, whereas the less conserved flanking regions may be important to modulate the affinities of interaction. Here, the less conserved L2 residues 189–220 or the less conserved SUMO-interacting motif IVAL286 may be important. The fact that infectivity and nuclear import were less affected for L2 IVAL286AAAA than RTR313EEE virions support an accessory rather than essential role for the SUMO-interacting motif. Unfortunately, we are precluded from further experimental validation of these hypotheses as long as the cellular interaction partners of L2 for chromosomal association remain elusive.

With this study, we propose a unified model for all PVs, where membrane translocation and nuclear entry of the L2/vDNA complex occur simultaneously in dividing cells by tethering of vDNA to mitotic chromosomes through L2 upon NEBD. This complex would remain associated with chromosomes throughout mitosis, and is thus directed into the nascent nuclei during cell division. L2 and the vDNA are recruited to prometaphase chromosomes through the interaction of a dedicated L2 CBR (residues 188–334 for HPV16 L2) with a mitosis-specific chromosomal cellular protein. The affinity of L2 to a common cellular protein may be PV type dependent, or different PV may engage different cellular targets. After mitosis, the subviral complex would have to be released from the chromosomes either upon dissociation of the cellular tethering factor from chromosomes, or upon high affinity binding to another nuclear factor that would direct the viral genome, for example, to PML-NBs [45, 46, 67]. In line with the latter possibility of regulated interactions, mitotic chromosomal association of HPV16 L2-EGFP was increased in the absence of, for example, the C-terminal PML-NB localization domain (Fig 2). The identification of the CBR of L2 for tethering of vDNA to mitotic chromatin now allows a more targeted search for a cellular recruiting factor. Moreover, the observed differences between PV types in their binding pattern to mitotic chromatin prompt future studies on the functional significance of these differences and on how they occur.

Importantly, our understanding of the dynamic interactions of the L2/vDNA complex has been inferred from steady state images of incoming virions and of live cell imaging from exogenously expressed L2. However, to fully understand the mechanistic dynamics of these processes, live cell imaging will be required. Since live cell imaging of this subviral complex is currently technically impossible, future efforts will aim at developing methods that allow dynamic analysis of incoming L2/vDNA.

## Materials and methods

All experimental results are derived from at least three independent experiments if not otherwise stated. Images were processed for presentation purposes using ImageJ (version 1.49m, National Institutes of Health, USA).

### Cell lines

HeLa cells were from ATCC. HeLa H2B-mCherry cells were a gift of D. Gerlich, Vienna [68]. Cells were maintained in DMEM (Sigma Aldrich) supplemented with 10% fetal calf serum (Merck Millipore). Medium was supplemented with 500 mg/ml G418 for HeLa H2B-mCherry cells. HaCaT NES-GFP-BAP cells were generated from HaCaT cells [69] and cultured as described in the accompanying report [47].

### Plasmid constructions

HPV16 L2 deletion mutants were constructed either by inverse PCR (L2(13–473), L2(1–220), L2(1–350), L2(1–140), L2(356–473)) or conventional cloning. For the inverse PCR, deletion was achieved by using primer pairs flanking the deleted region for outward PCR amplification from the HPV16 L2-EGFP plasmid (EF1alpha promoter-driven; generated by Chris Buck, provided by Martin Müller). The amplified products were digested with DpnI at 37°C for 1 h to remove the parental template before 5’-ends were phosphorylated and then self-ligated either at RT for 4 h or at 16°C overnight. In the conventional cloning approach, other HPV16 L2 deletion mutants were constructed by replacing the L2 gene in the HPV16 L2-EGFP plasmid between NotI and NheI sites with the coding sequence for the respective amino acid region. The insert was prepared in two steps: First, a DNA fragment was amplified from the HPV16 L2-EGFP plasmid with a forward Kozak consensus sequence-containing primer and a reverse NheI site-containing primer annealing to the desired N-terminal and C-terminal amino acid positions, respectively. Second, the obtained product was extended at the 5’-end by a NotI site-containing primer in a PCR with the previous reverse primer before digestion by NotI and NheI. The coding sequence for L2 of HPV5 was amplified from p5sheLL [70] and cloned into NotI/NheI-cleaved HPV16 L2-EGFP as above.

To construct HPV16 L2(269–334)-EGFP, the DNA fragment comprising the Kozak consensus sequence, the coding region for amino acids 269–334 and additional linker residues SGG was released by NotI/NheI digestion from a plasmid containing the synthetically produced insert (Eurofins). The DNA fragment was cloned into NotI/NheI-cleaved HPV16 L2-EGFP plasmid as described above.

To clone the coding sequence for L2 of HPV18 and MnPV into NotI/NheI-cleaved HPV16 L2-EGFP plasmid, internal NheI and NotI restriction sites in the respective L2 DNA sequences were mutated in the template plasmids HPV18L2h [71] and pJET-MnPVL2hum (provided by Frank Rösl, DKFZ) by site-directed mutagenesis before conventional cloning was performed as described above.

To generate BPV1 L2-EGFP, the L2 gene of BPV1 was amplified from pSheLL [72] with forward and reverse primers carrying KpnI and XmaI sites, respectively, and cloned into pEGFP-N3 (CMV promoter-driven) between KpnI and XmaI sites.

All point mutations were introduced with mutagenic primers by using the QuikChange II XL Site-Directed Mutagenesis Kit (Agilent Technologies) according to the manufacturer’s instructions. The HPV16 L2-EGFP plasmid was used as template for generating the point mutants L2(IVAL286AAAA)-EGFP, L2(RR297EE)-EGFP, L2(RR302/5AA)-EGFP and L2(RTR313EEE)-EGFP. p16SheLL [70] was used as template for introducing the point mutations encoding L2(RTR313EEE) and L2(IVAL286AAAA).

The sequences of primers that were employed in PCRs are listed in S1–S3 Tables. Sequences of obtained expression constructs were verified by Sanger sequencing.

### Viruses

Preparations of HPV16 PsVs containing GFP reporter plasmid or 5-ethynyl-2’-deoxyuridine (EdU)-labeled- DNA were performed as previously described using the plasmids p16SheLL and pCIneo-GFP [73, 74]. HPV16-L2(RTR313EEE) and HPV16-L2(IVAL286AAAA) PsVs were prepared like wild-type HPV16 PsVs but using p16SheLL mutated in the L2 gene. L2-BirA particles encapsidating a luciferase expression plasmid were generated by calcium phosphate transfection and CsCl purification as described in accompanying manuscript [47].

### Infectivity assay

5x10^4^ HeLa or HaCaT cells were plated in 12-well plates one day prior to infection with HPV16 to result in about 20% infection 48 h p.i. and with comparable amounts of HPV16-L2(RTR313EEE) or HPV16-L2(IVAL286AAAA). Additionally, high virus amounts were used to be able to detect any possible residual infectivity for the mutant virus. The inoculum was exchanged at 2 h p.i. with fresh medium. Cells were fixed 48 h p.i. in 4% PFA, and infectivity was assessed by flow cytometry (BD FACSCalibur) for GFP expression.

### Subcellular localization of vDNA in infected cells

To analyze vDNA of incoming HPV16 PsVs, 3x10^4^ HeLa Kyoto cells or HaCaT cells were seeded on glass cover slips one day prior to infection with either EdU-HPV16, EdU-HPV16-L2(RTR313EEE), or L2(IVAL286AAAA) PsVs. To determine trafficking of PsVs to the TGN under conditions, where nuclear entry is blocked by interphase arrest, cells were pretreated after adhesion with 15 μM aphidicolin (Sigma) for 16 h prior to infection, and infected in the presence of the inhibitor. Cells were fixed 20 h p.i. in 4% PFA, and EdU incorporated into the vDNA was detected using the Click-iT EdU Alexa Fluor 488 Imaging Kit (Invitrogen). After the EdU-Click-iT reaction, nuclei were visualized by staining with Hoechst 33258. Confocal image slices were acquired at the Zeiss LSM 780 confocal microscope with a 63x objective. Intensity-based colocalization analysis using the ImarisColoc module (Imaris, Bitplane).

For the colocalization study of vDNA with the TGN cells were immunostained with an antibody against the TGN marker p230 (BD Biosciences #611280, 1:1000 dilution), and a z interval of 0.8 μm was chosen for z-stacks covering the height of the interphase nucleus. Intensity-based colocalization analysis using the ImarisColoc module was performed to analyze localization of vDNA to the nucleus or TGN within three medial slices per cell. 10 to 20 cells were analyzed per condition in each experiment.

For studying colocalization of vDNA with mitotic chromosomes (in metaphase and anaphase cells) a z interval of 0.6 μm was chosen for z-stacks covering the height of the chromosome bulk. Colocalization of the vDNA with mitotic chromosomes was analyzed within the entire z-stack using ImarisColoc. 10 to 20 cells were analyzed in each experiment.

### Subcellular localization of L2 in infected cells

To analyze the L2 of incoming HPV16 PsVs, 3x10^4^ HeLa cells or HaCaT cells were seeded on glass cover slips one day prior to infection with either HPV16 or HPV16-L2(RTR313EEE) PsVs. Cells were fixed 20 h p.i. in 4% PFA, and L2 protein was detected using the L2 antibody K1L2 [75]. After the antibody staining, nuclei were visualized by staining with Hoechst 33258. Confocal image slices were acquired at the Zeiss LSM 780 confocal microscope with a 63x objective. Intensity-based colocalization analysis using the ImarisColoc module (Imaris, Bitplane).

For studying colocalization of L2 with mitotic chromosomes (in metaphase and anaphase cells) an interval of 0.6 μm was chosen for z-stacks covering the height of the chromosome bulk. Colocalization of the vDNA with mitotic chromosomes was analyzed within the entire z-stack using ImarisColoc. 10 to 20 cells were analyzed in each experiment.

### Electron microscopy

1-5x10^6^ purified HPV16 or HPV16-L2(RTR313EEE) PsVs in PBS/0.8 M NaCl were absorbed for 1 min on formvar-coated, carbon-sputtered grids. Particles were contrasted for 4 min with 1% phosphotungstic acid, pH 7.2, and then for 10 s with 1% uranylacetate/ddH2O. Directly after drying, samples were analyzed at 80 kV on a FEI-Tecnai 12 electron microscope (FEI, Eindhoven, Netherlands). Photographs of selected areas were documented with Olympus Veleta 4k CCD camera.

### SDS-polyacrylamide gel electrophoresis and Coomassie-staining

Similar amounts of purified HPV16 and HPV16-L2(RTR313EEE) PsVs were subjected to electrophoretic separation in a 12% SDS-polyacrylamide gel and Coomassie-staining. The relative amounts of L1, L2, and cellular histones derived from the encapsidated chromatinized reporter plasmid in virions were determined by densitometric analysis.

### Fluorescence microscopy analysis of HPV16 L2-EGFP during mitotic stages

To investigate the subcellular localization of HPV16 L2 in cells during the different mitotic stages, 5x10^4^ HeLa H2B-mCherry cells were plated on glass cover slips in 12-well plates, and transfected with a HPV16 L2-EGFP expression plasmid the next day using Lipofectamine2000 (Invitrogen). 9 h post transfection, cells were incubated with 2 mM thymidine for a single thymidine block for 15 h. When cells reached the first peak of mitosis 9–10 h after washing out the thymidine, they were fixed for 20 min in 4% PFA. Single slice images of transfected cells were acquired at the spinning disc microscope (Zeiss Axio Observer Z1, equipped with a Yokogawa CSU22 spinning disc module; Visitron Systems GmbH) with a 63x objective.

### Chromosomal association assay of PV L2-EGFP in prometaphase cells

To analyze the chromosomal association of PV L2-EGFP in prometaphase cells, 5000 HeLa H2B-mCherry cells or 7000 HaCaT cells were plated per well on optical 96-well plates one day prior to transfection with PV L2-EGFP constructs or EGFP expression plasmid as control. 24 h after transfection, cells were treated with 100 ng/ml nocodazole for 16 h in order to enrich cells in prometaphase. Then, cells were fixed for 30 min by carefully adding 12% PFA to the medium in the wells to yield a final concentration of 4%. The actin cytoskeleton was stained using Alexa Fluor 647 Phalloidin in PHEM Triton buffer (60 mM PIPES [piperazine-N,N′-bis(2-ethanesulfonic acid)], 10 mM EGTA, 2 mM MgCl_2_, 25 mM HEPES, pH 6.9, 0.1% Triton X-100). Single slice images were acquired at the spinning disc microscope with a 40x objective and analyzed with the cell image analysis software CellProfiler (version 2.1.1.). The chromosome and respective cellular area were segmented based on the H2B-mCherry (HeLa H2B-mCherry) or RedDot2 (HaCaT) and actin signal, respectively. The cytoplasmic area was determined by creating an inverted mask of the chromosome within the plasma membrane border. Due to differences in expression levels between the constructs, cells were classified based on their total EGFP intensities. Only cells with expression levels at which full-length HPV16 L2-EGFP showed association with mitotic chromosomes were quantified. The degree of chromosomal association was assessed by chromosomal association index (CAI), i.e. the ratio of mean EGFP fluorescence intensity (intensity per area) of chromosomally associated L2 over cytoplasmic L2 normalized by subtracting the median ratio for EGFP. If not otherwise stated, at least 50 cells per construct were analyzed this way from one to two experiments.

### Phylogenetic tree

The alignment of the L2 amino acid sequences of HPV16, 18, 5, BPV1 and MnPV by Clustal Omega (Sievers, 2011) was used to determine the evolutionary relationships with the approximate Likelihood-Ratio Test in PhyML 3.0 [76]. The phylogram was visualized with TreeDyn [77]. The branch length represents the amount of genetic changes in substitutions per site.

### PV L2 amino acid conservation analysis

The L2 amino acid sequences of HPV16, 18, 5, BPV1 and MnPV were analyzed for amino acid conservation by homology-extended multiple sequence alignment strategy in PRALINE [78]. The output scores from 0 for the least conserved alignment position, up to 10 for the most conserved alignment position were binned into five new color-coded categories: no (0–1, white), low (2–3, blue), intermediate low (4–5, green), intermediate high (6–7, orange), and high (8–10, red) conservation.

### L2-BirA experiments

As described in [47], 50,000 HaCaT NES-GFP-BAP cells were seeded in 24 wells plates one day prior to infection. To assay infectivity, the cells were infected with L2-BirA PsV at 2 x 10^8^ viral genomes/well. At 24 h p.i., the cells were washed with PBS and lysed in reporter lysis buffer from Promega (E3971). Luciferase activity was measured on a Beckman Coulter DTX-800 multimode plate reader using luciferase assay reagent from Promega (E4550) according to the manufacturer’s instructions. Luciferase signal was normalized to GAPDH signal from western blots of infected cell lysates. To assay translocation, the cells were infected with 150ng L1/well L2-BirA virus. At 24 h p.i., the cells were treated with pH 10.7 PBS for 2.5 minutes and then washed 2x with pH 7.2 PBS to remove non-internalized PsV. The cells were then lysed in 1X RIPA buffer (50 mM Tris-HCl pH 8.0, 150 mM NaCl, 1% NP40, 0.5% sodium deoxycholate, 0.1% SDS) supplemented with 1x reducing SDS-PAGE loading buffer, 1X protease inhibitor cocktail (Sigma P1860), and 1mM PMSF. The samples were then boiled for 5 minutes. After boiling, the samples were then resolved using SDS-PAGE and transferred onto a 0.45mm nitrocellulose membrane. The membrane was then cut in half at the 50kD marker. The upper half of the gel was blocked with 5% nonfat milk in TBST and stained with anti-L2 K4 at 1:5000. The lower half of the gel blocked in 100% Odyssey blocking buffer (Licor 927–40000) and stained with neutravidin DyLight 800 (Pierce 22853). The lower half of the blot was then reprobed sequentially with anti-GFP (Clontech 6323770) at 1:5000 and Goat anti-rabbit DyLight 680 (Pierce 35568) in 50% Odyssey blocker buffer/TBST. Blots were imaged using the Licor Odyssey Infrared Imaging System.

## Supporting information

S1 Table Primers used for generating HPV16 L2-EGFP constructs. (DOCX)

S2 Table Primers used for site-directed mutagenesis of HPV16 L2-EGFP. (DOCX)

S3 Table Primers used for generating PV L2-EGFP constructs. (DOCX)

S1 Fig Recruitment of L2-HA or L2-EGFP to mitotic chromatin in HeLa or HaCaT cells. (A) HeLa H2B-mCherry cells were transiently transfected with a HPV16 L2-3xHA expression plasmid. After cell fixations, high magnification images were acquired using a spinning disc microscope. (B) HaCaT cells were transiently transfected with HPV16 L2-EGFP, HPV16 L2(RTR313EEE)-EGFP, or HPV16 L2(IVAL286AAAA)-EGFP expression plasmid and stained for host chromosomes using RedDot2. Single confocal slices of full-length and mutant HPV16 L2-EGFP (left lane, green), chromosomes (center lane, red) and merges (right lane) are shown for representative cells. (C) The chromosomal association indices of L2-EGFPs were analyzed and plotted as in Fig 2D.
(TIF)

S2 Fig L2-EGFP chromosomal association assay. (A) Overview of the experimental setup. 24 h after transient transfection, HeLa H2B-mCherry cells were incubated overnight with 100 ng/ml nocodazole (noc.) prior to fixation, subsequent image acquisition by spinning disc microscopy, and computational image analysis. (B) Low magnification images of the histone marker show an efficient enrichment of prometaphase cells upon treatment with nocodazole (right) in comparison to untreated cells (left). Arrowheads indicate mitotic cells in the absence of nocodazole. (C, D) Depicted are high magnification single channel (EGFP (left, green), H2B-mCherry (center, red)) and merge images of cells transfected with HPV16 L2-EGFP (C), or the control EGFP (D). Note that L2-EGFP associated with the prometaphase chromosomes, while EGFP was excluded from the chromosomes.
(TIF)

S3 Fig Chromosomal association of further deletion mutants of HPV16 L2. Representative images of cells transfected with the remaining (A) N-terminally, (B) C-terminally, and (C) bilaterally truncated mutants of HPV16 L2-EGFP, which were quantitatively analyzed for their chromosomal association in Figs 2 and 3. Shown are HPV16 L2-EGFP (left column, green), H2B-mCherry (center column, red), and merges (right column).
(TIF)

S4 Fig Morphologic and biochemical analysis of HPV16 PsVs. (A) Morphologic analysis by EM of negatively stained HPV16 (left) and HPV16-L2(RTR313EEE) (right) PsVs. (B) Similar amounts of purified HPV16 and HPV16-L2(RTR313EEE) PsVs were subjected to SDS-PAGE and Coomassie-staining. The relative amounts of L1, L2, and cellular histones in virions were determined by densitometric analysis.
(TIF)

## References

[1] Chouhy D, Bolatti EM, Perez GR, Giri AA. Analysis of the genetic diversity and phylogenetic relationships of putative human papillomavirus types. The Journal of general virology. 2013;94(Pt 11):2480–8. doi: 10.1099/vir.0.055137-0 23997181 10.1099/vir.0.055137-0

[2] Schiffman M, Castle PE, Jeronimo J, Rodriguez AC, Wacholder S. Human papillomavirus and cervical cancer. Lancet. 2007;370(9590):890–907. doi: 10.1016/S0140-6736(07)61416-0 17826171 10.1016/S0140-6736(07)61416-0

[3] zur Hausen H. Papillomaviruses and cancer: from basic studies to clinical application. Nature reviews Cancer. 2002;2(5):342–50. doi: 10.1038/nrc798 12044010 10.1038/nrc798

[4] Cohen S, Au S, Pante N. How viruses access the nucleus. Biochim Biophys Acta. 2011;1813(9):1634–45. doi: 10.1016/j.bbamcr.2010.12.009 21167871 10.1016/j.bbamcr.2010.12.009

[5] Aydin I, Weber S, Snijder B, Samperio Ventayol P, Kuhbacher A, Becker M, et al Large scale RNAi reveals the requirement of nuclear envelope breakdown for nuclear import of human papillomaviruses. PLoS pathogens. 2014;10(5):e1004162 PubMed Central PMCID: PMC4038628. doi: 10.1371/journal.ppat.1004162 24874089 10.1371/journal.ppat.1004162 PMC4038628

[6] Pyeon D, Pearce SM, Lank SM, Ahlquist P, Lambert PF. Establishment of human papillomavirus infection requires cell cycle progression. PLoS pathogens. 2009;5(2):e1000318 Epub 2009/02/28. PubMed Central PMCID: PMC2642596. doi: 10.1371/journal.ppat.1000318 19247434 10.1371/journal.ppat.1000318 PMC2642596

[7] Egawa K. Do human papillomaviruses target epidermal stem cells? Dermatology. 2003;207(3):251–4. doi: 73085 14571065 10.1159/000073085

[8] Schmitt A, Rochat A, Zeltner R, Borenstein L, Barrandon Y, Wettstein FO, et al The primary target cells of the high-risk cottontail rabbit papillomavirus colocalize with hair follicle stem cells. Journal of virology. 1996;70(3):1912–22. PubMed Central PMCID: PMCPMC190020. 8627717 10.1128/jvi.70.3.1912-1922.1996 PMC190020

[9] Modis Y, Trus BL, Harrison SC. Atomic model of the papillomavirus capsid. The EMBO journal. 2002;21(18):4754–62. Epub 2002/09/18. PubMed Central PMCID: PMC126290. doi: 10.1093/emboj/cdf494 12234916 10.1093/emboj/cdf494 PMC126290

[10] Buck CB, Cheng N, Thompson CD, Lowy DR, Steven AC, Schiller JT, et al Arrangement of L2 within the Papillomavirus capsid. Journal of virology. 2008;82(11):5190–7. doi: 10.1128/JVI.02726-07 18367526 10.1128/JVI.02726-07 PMC2395198

[11] Howley PM. Papillomavirinae: the viruses and their replication In: Virology (2nd edition), Lippincott-Raven Publishers, Philadelphia, Pa. Fields Virology. 1996;2nd ed., 1996:2045–76.

[12] Meyers C, Frattini MG, Hudson JB, Laimins LA. Biosynthesis of human papillomavirus from a continuous cell line upon epithelial differentiation. Science. 1992;257(5072):971–3. Epub 1992/08/14. 1323879 10.1126/science.1323879

[13] Buck CB, Thompson CD, Pang YY, Lowy DR, Schiller JT. Maturation of papillomavirus capsids. Journal of virology. 2005;79(5):2839–46. Epub 2005/02/15. doi: 10.1128/JVI.79.5.2839-2846.2005 15709003 10.1128/JVI.79.5.2839-2846.2005 PMC548454

[14] Bienkowska-Haba M, Patel HD, Sapp M. Target cell cyclophilins facilitate human papillomavirus type 16 infection. PLoS pathogens. 2009;5(7):e1000524 Epub 2009/07/25. PubMed Central PMCID: PMC2709439. doi: 10.1371/journal.ppat.1000524 19629175 10.1371/journal.ppat.1000524 PMC2709439

[15] Cerqueira C, Liu Y, Kuhling L, Chai W, Hafezi W, van Kuppevelt TH, et al Heparin increases the infectivity of Human Papillomavirus type 16 independent of cell surface proteoglycans and induces L1 epitope exposure. Cellular microbiology. 2013;15(11):1818–36. Epub 2013/04/23. doi: 10.1111/cmi.12150 23601855 10.1111/cmi.12150 PMC4731924

[16] Cerqueira C, Samperio Ventayol P, Vogeley C, Schelhaas M. Kallikrein-8 proteolytically processes Human papillomaviruses in the extracellular space to facilitate entry into host cells. Journal of virology. 2015. 10.1128/JVI.00234-15 PMC4473586 25926655

[17] Culp TD, Budgeon LR, Christensen ND. Human papillomaviruses bind a basal extracellular matrix component secreted by keratinocytes which is distinct from a membrane-associated receptor. Virology. 2006;347(1):147–59. doi: 10.1016/j.virol.2005.11.025 16376962 10.1016/j.virol.2005.11.025

[18] Guan J, Bywaters SM, Brendle SA, Ashley RE, Makhov AM, Conway JF, et al Cryoelectron Microscopy Maps of Human Papillomavirus 16 Reveal L2 Densities and Heparin Binding Site. Structure. 2016. 10.1016/j.str.2016.12.001 28065506

[19] Richards RM, Lowy DR, Schiller JT, Day PM. Cleavage of the papillomavirus minor capsid protein, L2, at a furin consensus site is necessary for infection. Proceedings of the National Academy of Sciences of the United States of America. 2006;103(5):1522–7. doi: 10.1073/pnas.0508815103 16432208 10.1073/pnas.0508815103 PMC1360554

[20] Selinka HC, Florin L, Patel HD, Freitag K, Schmidtke M, Makarov VA, et al Inhibition of transfer to secondary receptors by heparan sulfate-binding drug or antibody induces noninfectious uptake of human papillomavirus. Journal of virology. 2007;81(20):10970–80. doi: 10.1128/JVI.00998-07 17686860 10.1128/JVI.00998-07 PMC2045555

[21] Raff AB, Woodham AW, Raff LM, Skeate JG, Yan L, Da Silva DM, et al The evolving field of human papillomavirus receptor research: a review of binding and entry. Journal of virology. 2013;87(11):6062–72. Epub 2013/03/29. PubMed Central PMCID: PMC3648114. doi: 10.1128/JVI.00330-13 23536685 10.1128/JVI.00330-13 PMC3648114

[22] Schelhaas M, Shah B, Holzer M, Blattmann P, Kuhling L, Day PM, et al Entry of human papillomavirus type 16 by actin-dependent, clathrin- and lipid raft-independent endocytosis. PLoS pathogens. 2012;8(4):e1002657 Epub 2012/04/27. PubMed Central PMCID: PMC3334892. doi: 10.1371/journal.ppat.1002657 22536154 10.1371/journal.ppat.1002657 PMC3334892

[23] Spoden G, Kuhling L, Cordes N, Frenzel B, Sapp M, Boller K, et al Human papillomavirus types 16, 18, and 31 share similar endocytic requirements for entry. Journal of virology. 2013;87(13):7765–73. Epub 2013/04/26. PubMed Central PMCID: PMC3700296. doi: 10.1128/JVI.00370-13 23616662 10.1128/JVI.00370-13 PMC3700296

[24] Day PM, Thompson CD, Schowalter RM, Lowy DR, Schiller JT. Identification of a role for the trans-Golgi network in human papillomavirus 16 pseudovirus infection. Journal of virology. 2013;87(7):3862–70. Epub 2013/01/25. PubMed Central PMCID: PMC3624235. doi: 10.1128/JVI.03222-12 23345514 10.1128/JVI.03222-12 PMC3624235

[25] Bienkowska-Haba M, Williams C, Kim SM, Garcea RL, Sapp M. Cyclophilins Facilitate Dissociation of the HPV16 Capsid Protein L1 from the L2/DNA Complex Following Virus Entry. Journal of virology. 2012. Epub 2012/07/05. 10.1128/JVI.00980-12 PMC3446629 22761365

[26] DiGiuseppe S, Bienkowska-Haba M, Hilbig L, Sapp M. The nuclear retention signal of HPV16 L2 protein is essential for incoming viral genome to transverse the trans-Golgi network. Virology. 2014;458–459:93–105. PubMed Central PMCID: PMCPMC4115330. 10.1016/j.virol.2014.04.024 PMC4115330 24928042

[27] Day PM, Roden RB, Lowy DR, Schiller JT. The papillomavirus minor capsid protein, L2, induces localization of the major capsid protein, L1, and the viral transcription/replication protein, E2, to PML oncogenic domains. Journal of virology. 1998;72(1):142–50. Epub 1998/01/07. PubMed Central PMCID: PMC109358. 9420209 10.1128/jvi.72.1.142-150.1998 PMC109358

[28] Becker KA, Florin L, Sapp C, Sapp M. Dissection of human papillomavirus type 33 L2 domains involved in nuclear domains (ND) 10 homing and reorganization. Virology. 2003;314(1):161–7. 14517069 10.1016/s0042-6822(03)00447-1

[29] Wang JW, Roden RB. L2, the minor capsid protein of papillomavirus. Virology. 2013;445(1–2):175–86. PubMed Central PMCID: PMCPMC3770800. doi: 10.1016/j.virol.2013.04.017 23689062 10.1016/j.virol.2013.04.017 PMC3770800

[30] Darshan MS, Lucchi J, Harding E, Moroianu J. The l2 minor capsid protein of human papillomavirus type 16 interacts with a network of nuclear import receptors. Journal of virology. 2004;78(22):12179–88. Epub 2004/10/28. PubMed Central PMCID: PMC525100. doi: 10.1128/JVI.78.22.12179-12188.2004 15507604 10.1128/JVI.78.22.12179-12188.2004 PMC525100

[31] Mamoor S, Onder Z, Karanam B, Kwak K, Bordeaux J, Crosby L, et al The high risk HPV16 L2 minor capsid protein has multiple transport signals that mediate its nucleocytoplasmic traffic. Virology. 2012;422(2):413–24. Epub 2011/12/14. PubMed Central PMCID: PMC3249505. doi: 10.1016/j.virol.2011.11.007 22154072 10.1016/j.virol.2011.11.007 PMC3249505

[32] Finnen RL, Erickson KD, Chen XS, Garcea RL. Interactions between papillomavirus L1 and L2 capsid proteins. Journal of virology. 2003;77(8):4818–26. Epub 2003/03/29. PubMed Central PMCID: PMC152166. doi: 10.1128/JVI.77.8.4818-4826.2003 12663788 10.1128/JVI.77.8.4818-4826.2003 PMC152166

[33] Klucevsek K, Daley J, Darshan MS, Bordeaux J, Moroianu J. Nuclear import strategies of high-risk HPV18 L2 minor capsid protein. Virology. 2006;352(1):200–8. doi: 10.1016/j.virol.2006.04.007 16733063 10.1016/j.virol.2006.04.007

[34] Okun MM, Day PM, Greenstone HL, Booy FP, Lowy DR, Schiller JT, et al L1 interaction domains of papillomavirus l2 necessary for viral genome encapsidation. Journal of virology. 2001;75(9):4332–42. Epub 2001/04/05. PubMed Central PMCID: PMC114178. doi: 10.1128/JVI.75.9.4332-4342.2001 11287582 10.1128/JVI.75.9.4332-4342.2001 PMC114178

[35] Roden RB, Day PM, Bronzo BK, Yutzy WHt, Yang Y, Lowy DR, et al Positively charged termini of the L2 minor capsid protein are necessary for papillomavirus infection. Journal of virology. 2001;75(21):10493–7. doi: 10.1128/JVI.75.21.10493-10497.2001 11581419 10.1128/JVI.75.21.10493-10497.2001 PMC114625

[36] Bergant M, Banks L. SNX17 facilitates infection with diverse papillomavirus types. Journal of virology. 2013;87(2):1270–3. Epub 2012/11/02. PubMed Central PMCID: PMC3554065. doi: 10.1128/JVI.01991-12 23115288 10.1128/JVI.01991-12 PMC3554065

[37] Pim D, Broniarczyk J, Bergant M, Playford MP, Banks L. A Novel PDZ Domain Interaction Mediates the Binding between Human Papillomavirus 16 L2 and Sorting Nexin 27 and Modulates Virion Trafficking. Journal of virology. 2015;89(20):10145–55. PubMed Central PMCID: PMCPMC4580170. doi: 10.1128/JVI.01499-15 26202251 10.1128/JVI.01499-15 PMC4580170

[38] Popa A, Zhang W, Harrison MS, Goodner K, Kazakov T, Goodwin EC, et al Direct binding of retromer to human papillomavirus type 16 minor capsid protein L2 mediates endosome exit during viral infection. PLoS pathogens. 2015;11(2):e1004699 PubMed Central PMCID: PMCPMC4334968. doi: 10.1371/journal.ppat.1004699 25693203 10.1371/journal.ppat.1004699 PMC4334968

[39] Bronnimann MP, Chapman JA, Park CK, Campos SK. A transmembrane domain and GxxxG motifs within L2 are essential for papillomavirus infection. Journal of virology. 2013;87(1):464–73. Epub 2012/10/26. PubMed Central PMCID: PMC3536380. doi: 10.1128/JVI.01539-12 23097431 10.1128/JVI.01539-12 PMC3536380

[40] Kamper N, Day PM, Nowak T, Selinka HC, Florin L, Bolscher J, et al A membrane-destabilizing peptide in capsid protein L2 is required for egress of papillomavirus genomes from endosomes. Journal of virology. 2006;80(2):759–68. Epub 2005/12/28. PubMed Central PMCID: PMC1346844. doi: 10.1128/JVI.80.2.759-768.2006 16378978 10.1128/JVI.80.2.759-768.2006 PMC1346844

[41] DiGiuseppe S, Keiffer TR, Bienkowska-Haba M, Luszczek W, Guion LG, Muller M, et al Topography of the Human Papillomavirus Minor Capsid Protein L2 during Vesicular Trafficking of Infectious Entry. Journal of virology. 2015;89(20):10442–52. PubMed Central PMCID: PMCPMC4580179. doi: 10.1128/JVI.01588-15 26246568 10.1128/JVI.01588-15 PMC4580179

[42] DiGiuseppe S, Luszczek W, Keiffer TR, Bienkowska-Haba M, Guion LG, Sapp MJ. Incoming human papillomavirus type 16 genome resides in a vesicular compartment throughout mitosis. Proceedings of the National Academy of Sciences of the United States of America. 2016;113(22):6289–94. doi: 10.1073/pnas.1600638113 27190090 10.1073/pnas.1600638113 PMC4896702

[43] Florin L, Becker KA, Lambert C, Nowak T, Sapp C, Strand D, et al Identification of a dynein interacting domain in the papillomavirus minor capsid protein l2. Journal of virology. 2006;80(13):6691–6. Epub 2006/06/16. PubMed Central PMCID: PMC1488977. doi: 10.1128/JVI.00057-06 16775357 10.1128/JVI.00057-06 PMC1488977

[44] Schneider MA, Spoden GA, Florin L, Lambert C. Identification of the dynein light chains required for human papillomavirus infection. Cellular microbiology. 2011;13(1):32–46. Epub 2010/12/21. doi: 10.1111/j.1462-5822.2010.01515.x 21166973 10.1111/j.1462-5822.2010.01515.x

[45] Bund T, Spoden GA, Koynov K, Hellmann N, Boukhallouk F, Arnold P, et al An L2 SUMO interacting motif is important for PML localization and infection of human papillomavirus type 16. Cellular microbiology. 2014;16(8):1179–200. doi: 10.1111/cmi.12271 24444361 10.1111/cmi.12271

[46] Day PM, Baker CC, Lowy DR, Schiller JT. Establishment of papillomavirus infection is enhanced by promyelocytic leukemia protein (PML) expression. Proceedings of the National Academy of Sciences of the United States of America. 2004;101(39):14252–7. Epub 2004/09/24. PubMed Central PMCID: PMC521143. doi: 10.1073/pnas.0404229101 15383670 10.1073/pnas.0404229101 PMC521143

[47] Calton CM, Bronnimann MP, Manson AR, Li S, Chapman JA, Suarez-Berumen M, et al Translocation of the Papillomavirus L2/vDNA Complex Across the Limiting Membrane Requires the Onset of Mitosis. PLoS pathogens. 13(5): e1006200. 10.1371/journal.ppat.1006200 PMC5412990 28463988

[48] Harper JV. Synchronization of cell populations in G1/S and G2/M phases of the cell cycle. Methods Mol Biol. 2005;296:157–66. 15576930 10.1385/1-59259-857-9:157

[49] Woodham AW, Da Silva DM, Skeate JG, Raff AB, Ambroso MR, Brand HE, et al The S100A10 subunit of the annexin A2 heterotetramer facilitates L2-mediated human papillomavirus infection. PloS one. 2012;7(8):e43519 Epub 2012/08/29. PubMed Central PMCID: PMC3425544. doi: 10.1371/journal.pone.0043519 22927980 10.1371/journal.pone.0043519 PMC3425544

[50] Bergant Marusic M, Ozbun MA, Campos SK, Myers MP, Banks L. Human papillomavirus L2 facilitates viral escape from late endosomes via sorting nexin 17. Traffic. 2012;13(3):455–67. Epub 2011/12/14. PubMed Central PMCID: PMC3276720. doi: 10.1111/j.1600-0854.2011.01320.x 22151726 10.1111/j.1600-0854.2011.01320.x PMC3276720

[51] Lipovsky A, Popa A, Pimienta G, Wyler M, Bhan A, Kuruvilla L, et al Genome-wide siRNA screen identifies the retromer as a cellular entry factor for human papillomavirus. Proceedings of the National Academy of Sciences of the United States of America. 2013;110(18):7452–7. Epub 2013/04/10. PubMed Central PMCID: PMC3645514. doi: 10.1073/pnas.1302164110 23569269 10.1073/pnas.1302164110 PMC3645514

[52] Bossis I, Roden RB, Gambhira R, Yang R, Tagaya M, Howley PM, et al Interaction of tSNARE syntaxin 18 with the papillomavirus minor capsid protein mediates infection. Journal of virology. 2005;79(11):6723–31. PubMed Central PMCID: PMCPMC1112158. doi: 10.1128/JVI.79.11.6723-6731.2005 15890910 10.1128/JVI.79.11.6723-6731.2005 PMC1112158

[53] Laniosz V, Nguyen KC, Meneses PI. Bovine papillomavirus type 1 infection is mediated by SNARE syntaxin 18. Journal of virology. 2007;81(14):7435–48. PubMed Central PMCID: PMCPMC1933340. doi: 10.1128/JVI.00571-07 17475643 10.1128/JVI.00571-07 PMC1933340

[54] Yang R, Yutzy WHt, Viscidi RP, Roden RB. Interaction of L2 with beta-actin directs intracellular transport of papillomavirus and infection. The Journal of biological chemistry. 2003;278(14):12546–53. Epub 2003/02/01. doi: 10.1074/jbc.M208691200 12560332 10.1074/jbc.M208691200

[55] Beckett D, Kovaleva E, Schatz PJ. A minimal peptide substrate in biotin holoenzyme synthetase-catalyzed biotinylation. Protein science: a publication of the Protein Society. 1999;8(4):921–9. PubMed Central PMCID: PMCPMC2144313. 10211839 10.1110/ps.8.4.921 PMC2144313

[56] Aydin I, Schelhaas M. Viral Genome Tethering to Host Cell Chromatin: Cause and Consequences. Traffic. 2016;17(4):327–40. doi: 10.1111/tra.12378 26787361 10.1111/tra.12378

[57] Bordeaux J, Forte S, Harding E, Darshan MS, Klucevsek K, Moroianu J. The l2 minor capsid protein of low-risk human papillomavirus type 11 interacts with host nuclear import receptors and viral DNA. Journal of virology. 2006;80(16):8259–62. Epub 2006/07/29. PubMed Central PMCID: PMC1563822. doi: 10.1128/JVI.00776-06 16873281 10.1128/JVI.00776-06 PMC1563822

[58] Fay A, Yutzy WHt, Roden RB, Moroianu J. The positively charged termini of L2 minor capsid protein required for bovine papillomavirus infection function separately in nuclear import and DNA binding. Journal of virology. 2004;78(24):13447–54. PubMed Central PMCID: PMCPMC533947. doi: 10.1128/JVI.78.24.13447-13454.2004 15564455 10.1128/JVI.78.24.13447-13454.2004 PMC533947

[59] Mallon RG, Wojciechowicz D, Defendi V. DNA-binding activity of papillomavirus proteins. Journal of virology. 1987;61(5):1655–60. PubMed Central PMCID: PMCPMC254149. 3033292 10.1128/jvi.61.5.1655-1660.1987 PMC254149

[60] Linding R, Jensen LJ, Diella F, Bork P, Gibson TJ, Russell RB. Protein disorder prediction: implications for structural proteomics. Structure. 2003;11(11):1453–9. 14604535 10.1016/j.str.2003.10.002

[61] Barbera AJ, Chodaparambil JV, Kelley-Clarke B, Joukov V, Walter JC, Luger K, et al The nucleosomal surface as a docking station for Kaposi's sarcoma herpesvirus LANA. Science. 2006;311(5762):856–61. doi: 10.1126/science.1120541 16469929 10.1126/science.1120541

[62] Matsumura S, Persson LM, Wong L, Wilson AC. The latency-associated nuclear antigen interacts with MeCP2 and nucleosomes through separate domains. Journal of virology. 2010;84(5):2318–30. PubMed Central PMCID: PMCPMC2820923. doi: 10.1128/JVI.01097-09 20032179 10.1128/JVI.01097-09 PMC2820923

[63] Barbera AJ, Ballestas ME, Kaye KM. The Kaposi's sarcoma-associated herpesvirus latency-associated nuclear antigen 1 N terminus is essential for chromosome association, DNA replication, and episome persistence. Journal of virology. 2004;78(1):294–301. PubMed Central PMCID: PMCPMC303411. doi: 10.1128/JVI.78.1.294-301.2004 14671111 10.1128/JVI.78.1.294-301.2004 PMC303411

[64] McBride AA. Replication and partitioning of papillomavirus genomes. Adv Virus Res. 2008;72:155–205. PubMed Central PMCID: PMCPMC3151303. doi: 10.1016/S0065-3527(08)00404-1 19081491 10.1016/S0065-3527(08)00404-1 PMC3151303

[65] You J, Croyle JL, Nishimura A, Ozato K, Howley PM. Interaction of the bovine papillomavirus E2 protein with Brd4 tethers the viral DNA to host mitotic chromosomes. Cell. 2004;117(3):349–60. 15109495 10.1016/s0092-8674(04)00402-7

[66] Poddar A, Reed SC, McPhillips MG, Spindler JE, McBride AA. The human papillomavirus type 8 E2 tethering protein targets the ribosomal DNA loci of host mitotic chromosomes. Journal of virology. 2009;83(2):640–50. PubMed Central PMCID: PMCPMC2612381. doi: 10.1128/JVI.01936-08 19004936 10.1128/JVI.01936-08 PMC2612381

[67] Florin L, Schafer F, Sotlar K, Streeck RE, Sapp M. Reorganization of nuclear domain 10 induced by papillomavirus capsid protein l2. Virology. 2002;295(1):97–107. Epub 2002/05/30. doi: 10.1006/viro.2002.1360 12033769 10.1006/viro.2002.1360

[68] Steigemann P, Wurzenberger C, Schmitz MH, Held M, Guizetti J, Maar S, et al Aurora B-mediated abscission checkpoint protects against tetraploidization. Cell. 2009;136(3):473–84. Epub 2009/02/11. doi: 10.1016/j.cell.2008.12.020 19203582 10.1016/j.cell.2008.12.020

[69] Boukamp P, Petrussevska RT, Breitkreutz D, Hornung J, Markham A, Fusenig NE. Normal keratinization in a spontaneously immortalized aneuploid human keratinocyte cell line. The Journal of cell biology. 1988;106(3):761–71. Epub 1988/03/01. PubMed Central PMCID: PMC2115116. 2450098 10.1083/jcb.106.3.761 PMC2115116

[70] Buck CB, Thompson CD, Roberts JN, Muller M, Lowy DR, Schiller JT. Carrageenan is a potent inhibitor of papillomavirus infection. PLoS pathogens. 2006;2(7):e69 Epub 2006/07/15. PubMed Central PMCID: PMC1500806. doi: 10.1371/journal.ppat.0020069 16839203 10.1371/journal.ppat.0020069 PMC1500806

[71] Pastrana DV, Buck CB, Pang YY, Thompson CD, Castle PE, FitzGerald PC, et al Reactivity of human sera in a sensitive, high-throughput pseudovirus-based papillomavirus neutralization assay for HPV16 and HPV18. Virology. 2004;321(2):205–16. Epub 2004/03/31. doi: 10.1016/j.virol.2003.12.027 15051381 10.1016/j.virol.2003.12.027

[72] Buck CB, Pastrana DV, Lowy DR, Schiller JT. Efficient intracellular assembly of papillomaviral vectors. Journal of virology. 2004;78(2):751–7. doi: 10.1128/JVI.78.2.751-757.2004 14694107 10.1128/JVI.78.2.751-757.2004 PMC368835

[73] Buck CB, Pastrana DV, Lowy DR, Schiller JT. Generation of HPV pseudovirions using transfection and their use in neutralization assays. Methods in molecular medicine. 2005;119:445–62. doi: 10.1385/1-59259-982-6:445 16350417 10.1385/1-59259-982-6:445

[74] Ishii Y, Tanaka K, Kondo K, Takeuchi T, Mori S, Kanda T. Inhibition of nuclear entry of HPV16 pseudovirus-packaged DNA by an anti-HPV16 L2 neutralizing antibody. Virology. 2010;406(2):181–8. Epub 2010/08/06. doi: 10.1016/j.virol.2010.07.019 20684966 10.1016/j.virol.2010.07.019

[75] Rubio I, Seitz H, Canali E, Sehr P, Bolchi A, Tommasino M, et al The N-terminal region of the human papillomavirus L2 protein contains overlapping binding sites for neutralizing, cross-neutralizing and non-neutralizing antibodies. Virology. 2011;409(2):348–59. Epub 2010/11/16. doi: 10.1016/j.virol.2010.10.017 21074234 10.1016/j.virol.2010.10.017

[76] Guindon S, Dufayard JF, Lefort V, Anisimova M, Hordijk W, Gascuel O. New algorithms and methods to estimate maximum-likelihood phylogenies: assessing the performance of PhyML 3.0. Syst Biol. 2010;59(3):307–21. doi: 10.1093/sysbio/syq010 20525638 10.1093/sysbio/syq010

[77] Chevenet F, Brun C, Banuls AL, Jacq B, Christen R. TreeDyn: towards dynamic graphics and annotations for analyses of trees. BMC Bioinformatics. 2006;7:439 PubMed Central PMCID: PMCPMC1615880. doi: 10.1186/1471-2105-7-439 17032440 10.1186/1471-2105-7-439 PMC1615880

[78] Simossis VA, Heringa J. PRALINE: a multiple sequence alignment toolbox that integrates homology-extended and secondary structure information. Nucleic Acids Res. 2005;33(Web Server issue):W289–94. PubMed Central PMCID: PMCPMC1160151. doi: 10.1093/nar/gki390 15980472 10.1093/nar/gki390 PMC1160151

