# Supplementary material for: A central region in the minor capsid protein of papillomaviruses facilitates viral genome tethering and membrane penetration for mitotic nuclear entry
Source: PLoS Pathog. 2017 May 2;13(5):e1006308. doi: 10.1371/journal.ppat.1006308 (PMC5412989; doi:10.1371/journal.ppat.1006308)
Supplement: S1 Table — (DOCX) [file ppat.1006308.s001.docx]

| **HPV16 L2 aa** | **Forward primer 5’- 3’** | **Reverse primer 5’- 3’** |
| --- | --- | --- |
| 13-473 | GCCAGCGCCACCCAGCTGTACAAGACC | CATGGTGGCTCTAGAGCGGCCGCGG |
| 141-473 | TCTAGAGCCACCATGACCACCCCCGCCATCC | ACTAGCTAGCGCGGCCAGGCTCACGTC |
| 221-473 | TCTAGAGCCACCATGCCCGTGGCCAGGCTG | ACTAGCTAGCGCGGCCAGGCTCACGTC |
| 356-473 | GCCAGCCCCACCAGCATCAACAACGGC | CATGGTGGCTCTAGAGCGGCCGCGG |
| 1-443 | TCTAGAGCCACCATGAGGCACAAGAGGAGC | ACTAGCTAGCGCGGCGTCGGCGATGATG |
| 1-350 | CTAGCGCTACCGGTCGCCACCATGG | GGTGTAGGTGCTGGGGGTGATGGTCTGC |
| 1-334 | TCTAGAGCCACCATGAGGCACAAGAGGAGC | ACTAGCTAGCGCGTCGATGGTGCTCAGG |
| 1-318 | TCTAGAGCCACCATGAGGCACAAGAGGAGC | ACTAGCTAGCGCCTTGCCGCTCCTGGTC |
| 1-220 | CTAGCGCTACCGGTCGCCACCATGG | CCTGCTGCCGGGGATGGGGGTGCTG |
| 1-140 | CTAGCGCTACCGGTCGCCACCATGG | GTCGGTGCTGGTGGTGATGCTGAAGCC |
| 81-350 | TCTAGAGCCACCATGACCGCCACCGACAC | ACTAGCTAGCGCGGTGTAGGTGCTGG |
| 141-355 | TCTAGAGCCACCATGACCACCCCCGCCATCC | ACTAGCTAGCGCGGCGTGGCTGGTGGTGG |
| 150-350 | TCTAGAGCCACCATGAACACCGTGACCAC | ACTAGCTAGCGCGGTGTAGGTGCTGG |
| 188-334 | TCTAGAGCCACCATGATCAGCACCCACAAC | ACTAGCTAGCGCGTCGATGGTGCTCAGG |
| 221-334 | TCTAGAGCCACCATGCCCGTGGCCAGGCTG | ACTAGCTAGCGCGTCGATGGTGCTCAGG |
| 188-318 | TCTAGAGCCACCATGATCAGCACCCACAAC | ACTAGCTAGCGCCTTGCCGCTCCTGGTC |
