# Supplementary material for: A central region in the minor capsid protein of papillomaviruses facilitates viral genome tethering and membrane penetration for mitotic nuclear entry
Source: PLoS Pathog. 2017 May 2;13(5):e1006308. doi: 10.1371/journal.ppat.1006308 (PMC5412989; doi:10.1371/journal.ppat.1006308)
Supplement: S2 Table — (DOCX) [file ppat.1006308.s002.docx]

| **HPV16 L2 aa** | **Forward primer 5’- 3’** | **Reverse primer 5’- 3’** |
| --- | --- | --- |
| IVAL286AAAA | GACTTCCTGGACGCCGCGGCCGCGCACAGGCCCG | CGGGCCTGTGCGCGGCCGCGGCGTCCAGGAAGTC |
| RR297EE | GCCCGCCCTGACCAGCGAGGAGACCGGCATCAGGTAC | GTACCTGATGCCGGTCTCCTCGCTGGTCAGGGCGGGC |
| RR302/5AA | GGAGGACCGGCATCGCGTACAGCGCGATCGGCAACAAGC | GCTTGTTGCCGATCGCGCTGTACGCGATGCCGGTCCTCC |
| RTR313EEE | GATCGGCAACAAGCAGACCCTGGAGGAGGAGAGCGGCAAGAGCATCGGCGCC | GGCGCCGATGCTCTTGCCGCTCTCCTCCTCCAGGGTCTGCTTGTTGCCGATC |
