# Supplementary material for: A central region in the minor capsid protein of papillomaviruses facilitates viral genome tethering and membrane penetration for mitotic nuclear entry
Source: PLoS Pathog. 2017 May 2;13(5):e1006308. doi: 10.1371/journal.ppat.1006308 (PMC5412989; doi:10.1371/journal.ppat.1006308)
Supplement: S3 Table — (DOCX) [file ppat.1006308.s003.docx]

| **PV L2** | **Forward primer 5’- 3’** | **Reverse primer 5’- 3’** |
| --- | --- | --- |
| HPV18 | TCTAGAGCCACCATGGTCAGCCATAGGGCTG | ACTAGCTAGCGCAGCGGCGACGAACCC |
| HPV5 | TGATGCGGCCGCTCTAGAGCCACCATG | ACTAGCTAGCGCGAGGTACTTGCGCTTCC |
| BPV1 | CGAGGTACCATGAGCGCCCGCAAGAGAG | ATTCCCGGGGCGGCATGCTTGCGCTTC |
| MnPV | TCTAGAGCCACCATGAGCCGCCGCCGCAAG | ACTAGCTAGCGCTGCGGCGAGCACCC |
