# Supplementary figures and images for: A central region in the minor capsid protein of papillomaviruses facilitates viral genome tethering and membrane penetration for mitotic nuclear entry
Source: PLoS Pathog. 2017 May 2;13(5):e1006308. doi: 10.1371/journal.ppat.1006308 (PMC5412989; doi:10.1371/journal.ppat.1006308)

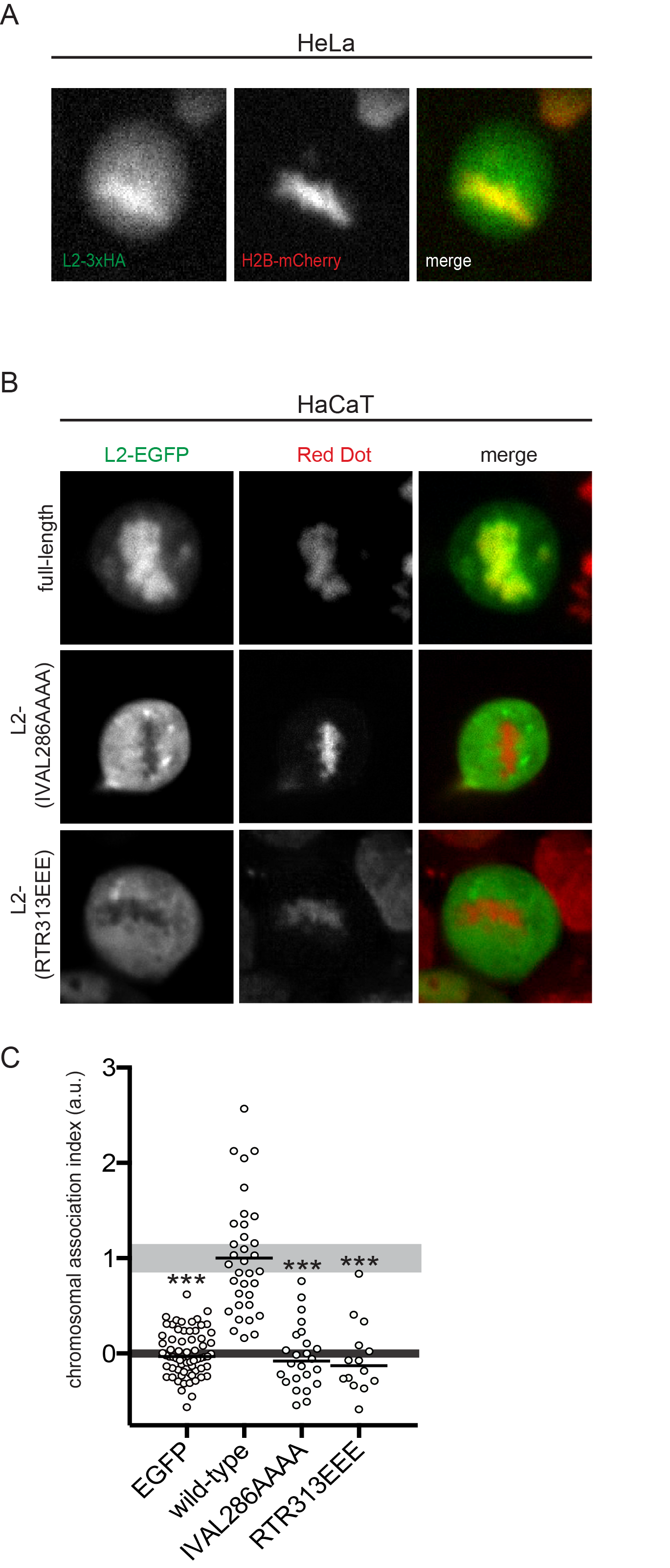

Supplement: S1 Fig — (A) HeLa H2B-mCherry cells were transiently transfected with a HPV16 L2-3xHA expression plasmid. After cell fixations, high magnification images were acquired using a spinning disc microscope. (B) HaCaT cells were transiently transfected with HPV16 L2-EGFP, HPV16 L2(RTR313EEE)-EGFP, or HPV16 L2(IVAL286AAAA)-EGFP expression plasmid and stained for host chromosomes using RedDot2. Single confocal slices of full-length and mutant HPV16 L2-EGFP (left lane, green), chromosomes (center lane, red) and merges (right lane) are shown for representative cells. (C) The chromosomal association indices of L2-EGFPs were analyzed and plotted as in Fig 2D. (TIF) [file ppat.1006308.s004.tif]

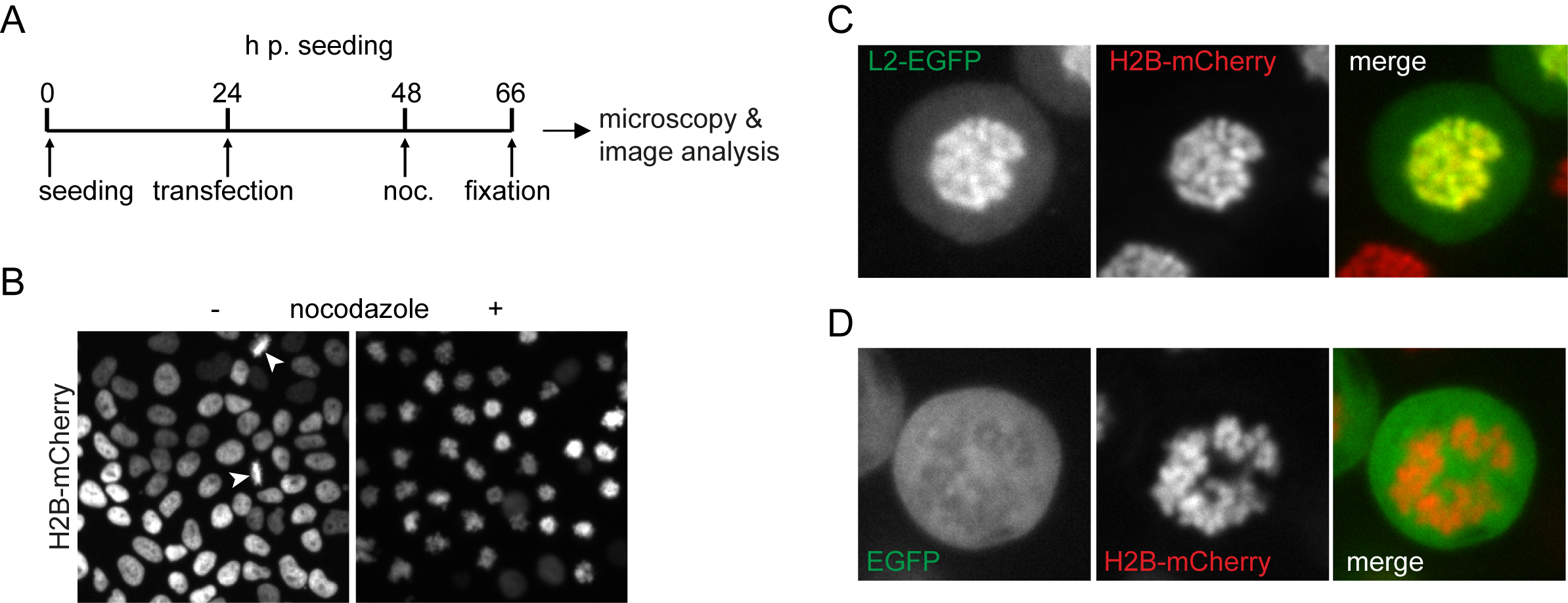

Supplement: S2 Fig — (A) Overview of the experimental setup. 24 h after transient transfection, HeLa H2B-mCherry cells were incubated overnight with 100 ng/ml nocodazole (noc.) prior to fixation, subsequent image acquisition by spinning disc microscopy, and computational image analysis. (B) Low magnification images of the histone marker show an efficient enrichment of prometaphase cells upon treatment with nocodazole (right) in comparison to untreated cells (left). Arrowheads indicate mitotic cells in the absence of nocodazole. (C, D) Depicted are high magnification single channel (EGFP (left, green), H2B-mCherry (center, red)) and merge images of cells transfected with HPV16 L2-EGFP (C), or the control EGFP (D). Note that L2-EGFP associated with the prometaphase chromosomes, while EGFP was excluded from the chromosomes. (TIF) [file ppat.1006308.s005.tif]

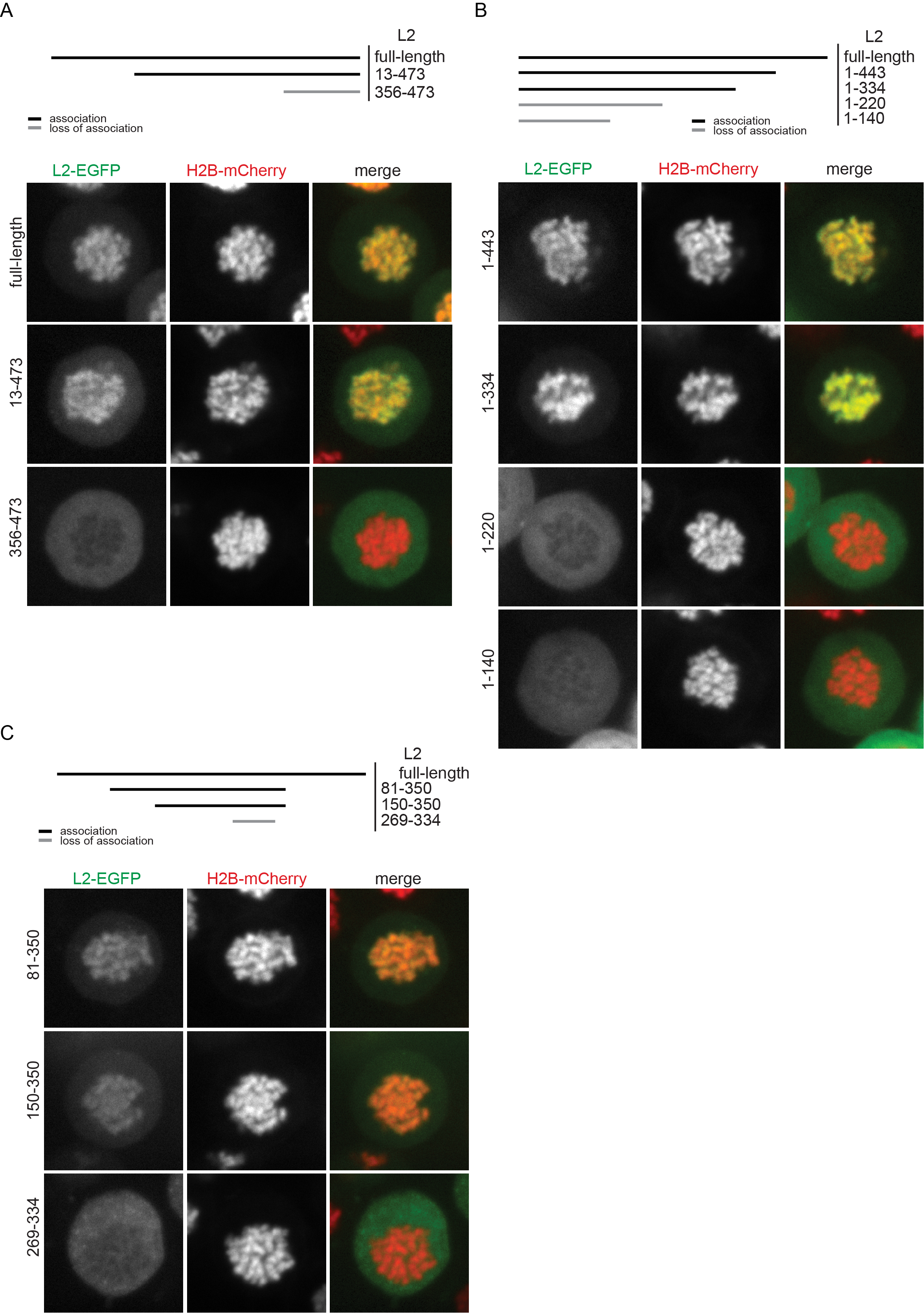

Supplement: S3 Fig — Representative images of cells transfected with the remaining (A) N-terminally, (B) C-terminally, and (C) bilaterally truncated mutants of HPV16 L2-EGFP, which were quantitatively analyzed for their chromosomal association in Figs 2 and 3. Shown are HPV16 L2-EGFP (left column, green), H2B-mCherry (center column, red), and merges (right column). (TIF) [file ppat.1006308.s006.tif]

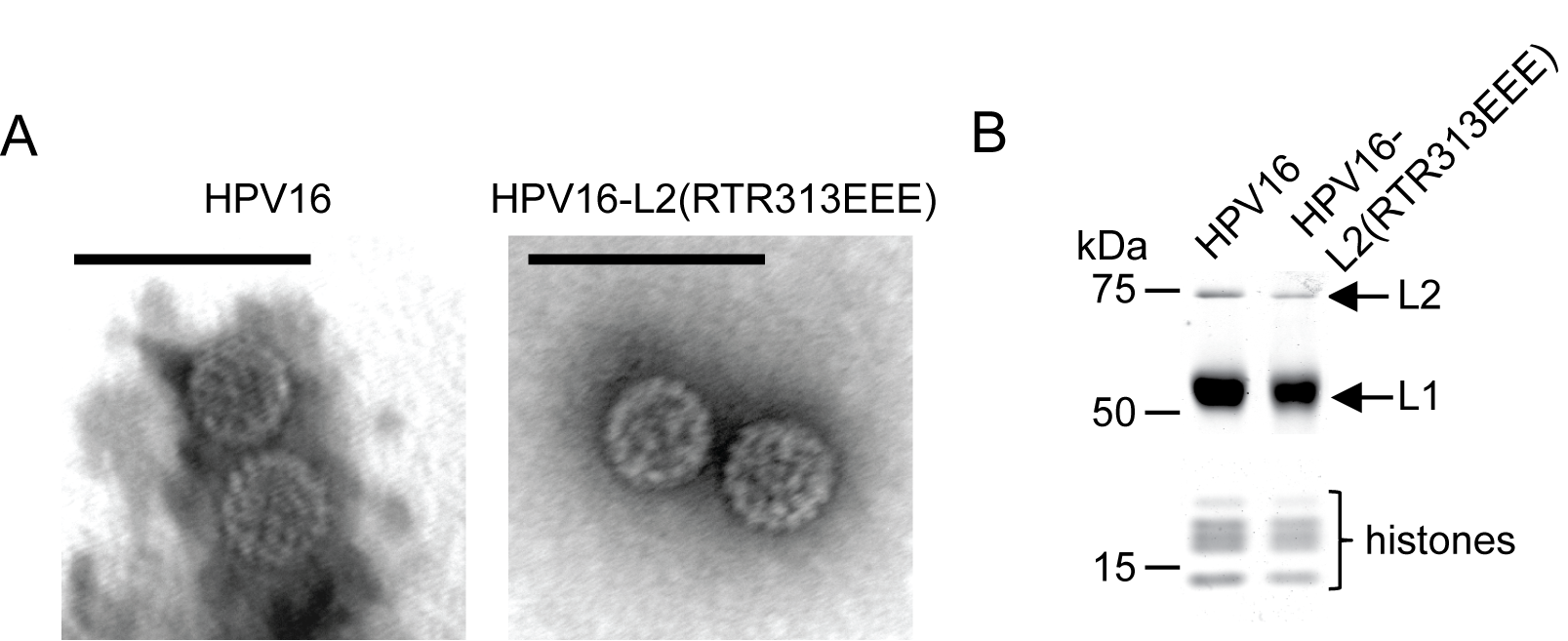

Supplement: S4 Fig — (A) Morphologic analysis by EM of negatively stained HPV16 (left) and HPV16-L2(RTR313EEE) (right) PsVs. (B) Similar amounts of purified HPV16 and HPV16-L2(RTR313EEE) PsVs were subjected to SDS-PAGE and Coomassie-staining. The relative amounts of L1, L2, and cellular histones in virions were determined by densitometric analysis. (TIF) [file ppat.1006308.s007.tif]
